# Barriers and enablers to using evidence-based antibiotic prescription guidelines in primary care: a qualitative systematic review and synthesis using the theoretical domains framework

**DOI:** 10.1186/s43058-025-00806-w

**Published:** 2026-02-16

**Authors:** Krystal Bursey, Andrea M. Patey, Holly Etchegary, Kris Aubrey-Bassler, Victoria Kavanagh, Andrea Pike, Kristen Romme, Jeremy M. Grimshaw, Amanda Hall

**Affiliations:** 1 Primary Healthcare Research Unit, Memorial University of Newfoundland, 300 Prince Philip Dr, St. John’s, Newfoundland and Labrador, NL A1B 3V6 Canada; 2 Clinical Epidemiology, Memorial University of Newfoundland, St. John’s, Newfoundland and Labrador, Canada; 3 Methodological and Implementation Research, Ottawa Hospital Research Institute, Ottawa, Canada; 4 Health Sciences Library, Memorial University of Newfoundland, St. John’s, Newfoundland and Labrador, Canada; 5 Department of Medicine, University of Ottawa, Ottawa, Canada

**Keywords:** Antibiotics, Upper respiratory tract infections, Qualitative, Primary care

## Abstract

**Background:**

Antibiotics are commonly overprescribed for upper respiratory tract infections (URTIs) in primary care against widely known guideline recommendations. To design an effective intervention to improve adherence to URTI guideline-based care, it is important to understand why the behaviour persists. This review aimed to conduct a qualitative systematic review of the barriers and enablers to URTI guideline-based prescribing for FPs in primary care using a Theoretical Domains Framework (TDF)-based analysis.

**Methods:**

The following databases were searched with no date or language restrictions from inception to December 2024: MEDLINE, Web of Science, CINAHL, Embase, The Cochrane Library, and APA PsycInfo. Qualitative studies that explored FP’s experiences with following antibiotic prescription guidelines for URTIs were included. Data on the barriers and enablers for URTI guideline adherence were extracted and analyzed using the TDF approach and categorized into the 14 TDF domains. Barriers and enablers were assessed for confidence using the Grading of Recommendations Assessment, Development and Evaluation—Confidence in the Evidence from Reviews of Qualitative Research (GRADE-CERQual) approach.

**Results:**

A total of 2837 articles were screened, and 23 studies were included. The included studies had moderate to high methodological rigour and included a total of 516 FPs. A total of 61 barriers across 13 TDF domains were identified but only 17 barriers across 8 TDF domains were determined to have high confidence. These barriers with high confidence largely centered on (1) lack of support for guideline-based prescribing and physician fatigue, (2) perception of patient demand for antibiotics and the doctor-patient relationship, and (3) poor physician understanding of antibiotic resistance and antibiotics and their role in patient care. A total of 40 enablers across 13 TDF domains were identified but only 10 enablers across 8 TDF domains were judged to have high confidence. The enablers with high confidence were related to (1) knowledge of the impact of antibiotic use on antibiotic resistance and prioritizing evidence-based care, and (2) antibiotic reduction strategies for both physician and patient.

**Conclusion:**

This review found 17 barriers to adhering to URTI antibiotic prescribing guidelines for FPs in primary care across 8 TDF domains. This is important because many interventions do not target all the TDF domains we identified as barriers. Future intervention design should consider adopting strategies to target these domains to ensure its efficacy in improving guidelines adherence.

**Supplementary Information:**

The online version contains supplementary material available at 10.1186/s43058-025-00806-w.

Contributions to the literature

- Our review is the first comprehensive review of the barriers and enablers to antibiotic prescribing for URTIs in primary care using a TDF-based analysis and GRADE CERQual synthesis.
- Our findings highlight underlying mechanisms of antibiotic overprescribing for URTIs that have yet to be addressed by interventions.
- The findings of this review can be used to develop theory-informed behaviour change interventions to improve antibiotic prescribing for URTIs.

## Introduction

Overprescribing antibiotics poses a significant threat to healthcare globally because of its contribution to antibiotic resistance [1–3]. While the overprescription of antibiotics has been reported in many areas of healthcare, it is most often reported in primary care. For example, it has been estimated that 92% of antibiotics in Canada are prescribed in non-hospital-based settings [4, 5]. This overprescription is likely due in large part to upper respiratory tract infections (URTIs), as they are among the most common conditions presented in primary care settings [5]. Numerous guidelines have been developed for the care and management of URTIs (and related conditions such as otitis media, pharyngitis, and sinusitis; e.g., the National Institute for Health and Care Excellence (NICE) guidelines and the Choosing Wisely Canada recommendations) [6–10]. All recommend prescribing antibiotics only when there are clear indications. For example, antibiotics are never indicated for conditions such as the common cold and bronchitis as these are caused by viral rather than bacterial infections [10–12]. Similarly, antibiotics are only indicated for pharyngitis, sinusitis, and otitis media if they meet specific criteria indicating a higher likelihood of bacterial infection [10, 13–15]. Despite these guideline recommendations, antibiotics continue to be prescribed for URTIs, with reported rates ranging from 15.4% to 83.7% [5, 16]. While reported rates vary, likely due to data gathering and measurement challenges, this variability also highlights the lack of consistency in guideline-based prescribing.

Significant resources and research have been dedicated to developing and implementing interventions to address antibiotic overprescribing. For example, two systematic reviews by Tonkin-Crine et al. and Spurling et al. of clinician-targeted interventions for reducing antibiotic prescribing for acute RTIs found that most interventions resulted in minor changes (less than 25% between groups), with only a few achieving greater than 60% reduction in antibiotic prescribing [17, 18]. While some interventions did achieve a significant reduction in antibiotic prescriptions, that may not accurately reflect whether guideline adherence or appropriate antibiotic prescribing improved. Furthermore, many of the interventions utilized strategies (e.g., diagnostic point-of-care testing) that would likely not be practical for most family physicians (FPs) as they are expensive, and FPs (or their patients) would likely have to pay for these tests themselves. Thus, there have been interventions developed that have resulted in some reductions in antibiotic prescribing. However, that does not necessarily indicate that antibiotic prescribing has improved and there still remains a need for improved intervention development and research to address antibiotic prescribing guideline adherence and antibiotic overprescribing for URTIs.

Recommendations for effective healthcare intervention design and development suggest that it is necessary to understand the problem by identifying factors that influence the targeted behaviour (i.e., prescribing antibiotics for URTIs) [19]. Additionally, the application of theory to the investigation of a targeted behaviour and the intervention development and design can greatly improve the chances of successful behaviour change [20–23]. One behavioural science framework commonly applied to understand healthcare providers' clinical practice behaviours is the Theoretical Domains Framework (TDF). The TDF is an established framework that has synthesized 38 behaviour change theories and 128 key theoretical constructs into 14 domains that provides a theoretical lens through which to view the cognitive, affective, social, and environmental influences on behaviour [22, 24]. These influences can then be targeted by domain-appropriate strategies, and an intervention may be designed with a better chance of successful practice behaviour change.

To date, there has been substantial qualitative work exploring the barriers and enablers for overprescribing antibiotics, and at least three systematic reviews have aimed to synthesize these data [25–27]. However, the most recent review only included studies up to 2016 [25]. Our review aimed to build upon the current literature by conducting an up-to-date qualitative systematic review of the barriers and enablers to URTI guideline-based prescribing for family physicians (FPs) in primary care using a TDF-based analysis. Results from this review can then be used to inform strategy selection for future intervention designs. Additionally, we aimed to understand if the barriers and enablers are generalizable across countries or if they are more contextually specific.

## Methods

The protocol for this review has been prospectively published in BMJ Open (https://bmjopen.bmj.com/content/12/11/e066681) [28]. Our protocol followed guidelines from the Preferred Reporting Items for Systematic Reviews and Meta-Analyses (PRISMA) statement and the JBI Manual for Evidence Synthesis for Systematic Reviews of Qualitative Evidence [29, 30].

### Searches

An experienced librarian developed a comprehensive search strategy for the following databases: MEDLINE (Ovid), Web of Science Core Collection, CINAHL Plus (EBSCOhost), Embase (Embase.com), The Cochrane Library (Wiley), and APA PsycInfo (EBSCOhost) [31]. See Additional file 1 for our search strategy. All databases were searched from inception to December 2024, with no date or language restrictions. All included studies' references were screened, and citations were tracked to identify additional papers not included in the database search. We also identified three previous relevant systematic reviews that were included in our reference screening and citation tracking [25–27].

### Study inclusion and exclusion criteria

We included articles that focused on (1) experiences with guideline-based antibiotic prescribing for URTIs, (2) with family physicians as study participants, (3) were conducted in primary care settings only, and (4) utilized a qualitative methodology. We updated our population inclusion criteria from the publication of our protocol to further elaborate on our screening process. For example, studies that examined “respiratory tract infections (RTIs)” were excluded if they did not identify which RTIs they included, and the authors could not be contacted for more clarification. See Table 1 for the inclusion and exclusion criteria.

**Table 1:** Inclusion and exclusion criteria by PICOS terms, languages, and publication

PICoS Term	Inclusion Criteria	Exclusion Criteria
Population	FPs discussing URTIs as defined by the Choosing Wisely Canada Guidelines: ◦ Otitis Media ◦ Pharyngitis ◦ Sinusitis ◦ Bronchitis ◦ The common cold	We excluded articles that only report on: ◦ Any other illness for which an antibiotic may be prescribed (e.g., lower respiratory infections, surgical site infections, infections of teeth/mouth) If the findings contained a mixture of conditions, we excluded them unless the results could be separated. If the article included “respiratory tract infections” or “lower respiratory tract infections” but were not clear in which conditions they included (and clarification could not be sought from the authors), they were excluded
Phenomenon of Interest	FPs prescribing antibiotics for URTIs	We excluded articles that only report on: ◦ Any other healthcare professional that can prescribe antibiotics (e.g., nurse practitioners, pharmacists, physicians of other specializations) If the findings contained a mixture of healthcare professionals, we excluded the study if the FPs results cannot be separately extracted or if > 25% of the sample contains non-FPs, as the data was then considered unrepresentative
Context	Patients of any age with URTIs in primary care settings	We excluded articles that only reported about: Patients with URTIs or any other infection or condition in hospital, outpatient (outside of primary care clinics) or ambulatory settings
Study Design	All types of primary qualitative studies (i.e., no reviews) and mixed method studies if sufficient qualitative data are provided (e.g. separate qualitative data analysis)	We excluded articles if they were: ◦ Single-case studies, quantitative studies, interventional studies, or studies that summarize the results of an original study
Languages	Any language	If an appropriate translator could not be found, the article was reported in the number of studies found but not included in the analysis. We considered Google translate insufficient to translate qualitative data as meaningful data may be lost or misinterpreted using unreliable translation methods
Type of Publication	Peer-reviewed journal articles	Book chapters, reviews, summaries, opinion pieces If the full text of an article is unavailable (and contact cannot be made to authors for a copy), data is unpublished or not peer-reviewed as the inclusion of these articles is considered controversial
Date of Publication	No restrictions	N/A

### Article screening

**Fig. 1:**
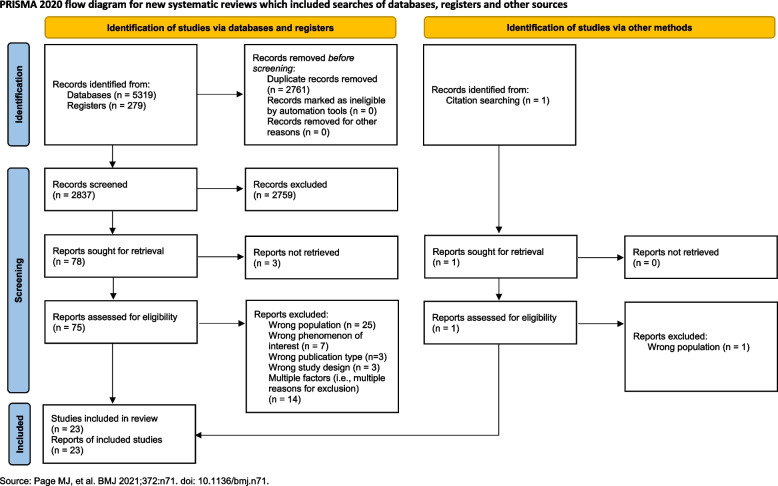
PRISMA flow diagram

All titles identified by the initial search were imported into Covidence systematic review software (available from covidence.org), and duplicates were removed [32]. Two reviewers (KB & VK) screened article titles and abstracts of all studies identified and completed the full-text review screening following a screening template with the pre-defined eligibility criteria. A third reviewer mediated disagreements if the reviewers could not reach a consensus. The screening process was documented using the PRISMA flow diagram (Fig. 1) [33].

### Study quality assessment (reporting and methodological rigour)

To assess methodological quality, we followed the process outlined by Hall et al. [34], which has combined elements from the Critical Appraisal Skills Program (CASP) [35] methodological section B on methods and the four methodological domains from the Consolidated Criteria for Reporting Qualitative Research (COREQ) [36] guidelines (Recruitment, Data Collection, Researcher-participant relationship, and Analysis). Two reviewers (KB & VK) applied this tool to each included study and scored each question in the checklist as "yes", "no", or "can't tell" and gave an overall score to determine if the study would be ranked as having good, moderate, or low methodological rigour. Any disagreements were resolved via consensus, and a third reviewer was consulted if necessary. The COREQ was used to assess reporting quality [36].

### Data extraction strategy

One researcher (KB) extracted all data following data extraction templates and another researcher (VK) reviewed the coding. Any discrepancies were resolved via consensus. Information extracted included study year, country, sample size, research aims, and data collection methods. Additionally, the results of the included studies were extracted in terms of the themes of the main findings.

### Data synthesis

#### Target behaviour

The target behaviour for this analysis was based on Choosing Wisely Canada’s evidence-based prescribing guidelines for family physicians – specifically, that antibiotics should not be prescribed for URTIs [10].

### TDF coding and domain-level synthesis

To synthesize the extracted data for this review, the TDF was used [24, 37]. Using the TDF approach involved identifying factors that influence the phenomenon of interest (i.e., FPs following guideline-based care for URTIs) from the results section of included studies and categorizing them as a barrier (i.e., preventing the guideline adherence or behaviour change) or enabler (i.e., facilitating guideline adherence). Then we deductively assigned the quotes and author-identified barriers and enablers to one or more TDF domains. For example, if a study reported that patient demand was a key factor influencing antibiotic prescribing behavior, this was coded as the Social Influences domain of the TDF. A codebook was created and informed by previous research conducted in the area. Under the direction of a TDF expert (AMP), one researcher with a psychology background (KB) was trained to code extracted data to the TDF domains using Google Sheets (available from https://docs.google.com/spreadsheets/). The TDF expert reviewed the coding for accuracy and appropriateness. The results were also the reviewed by several members of the patient and public advisory council of the provincial Support Unit, a group of 20 + members of the public with whom provincial research teams regularly engage. They received a summary of the barriers and enablers with high confidence and were asked to review these findings and provide feedback on our interpretation of the data and clarity of the findings. No significant changes were suggested by the council. The extracted data was analyzed to determine the number of contributing studies for each barrier and enabler, as well as to describe the relevant study information to prepare the data for the confidence assessment.

### Confidence assessment of findings

The Grading of Recommendations Assessment, Development and Evaluation—Confidence in the Evidence from Reviews of Qualitative Research (GRADE-CERQual) approach was used to determine how much confidence to place in each barrier and enabler identified from the TDF synthesis [38]. Identifying the barriers and enablers with high levels of confidence allowed us to determine which ones are likely a reasonable representation of the phenomenon of interest and should be targeted when developing future interventions to address the inappropriate prescribing of antibiotics for URTIs. Barriers and enablers with moderate confidence require more research before their impact on the target behaviour can be confirmed. Low/very low confidence levels indicate that these themes are not likely to represent barriers to following antibiotic prescribing guidelines globally and should not be addressed in future interventions [38].

Using the Interactive Summary of Qualitative Findings (iSoQ) software (available from https://isoq.epistemonikos.org/), each individual barrier and enabler was assessed on the four components of the GRADE-CERQual approach, and each barrier and enabler was given a rating of serious, moderate, minor or no/very minor concerns on each component. The four components are (1) the methodological rigour of the supporting studies (2), the relevance of the supporting studies to the review question (3), the coherence between the supporting data and the barrier or enabler, and (4) the adequacy of the supporting data for the barrier or enabler. Each barrier or enabler was then given an overall confidence level of high, moderate, low, or very low based on how well they were judged on each of the four components. Confidence levels start at high and then are downgraded if a component was judged to have serious or moderate concerns. This analysis was completed by a researcher (KB) in consultation with AH. Since the publication of the protocol, KB and AH have developed additional rules for specific issues that arose during the GRADE-CERQual analysis. These rules were for findings derived from a single study, and specific contextual issues like country-specific findings, findings related to a specific URTI, and findings related to a specific primary care funding model. For more information on these additional rules, see additional file 2.

### Overarching synthesis (across domains)

To summarize our main findings, we took the barriers and enablers from the TDF domain level synthesis that were judged to have high confidence according to the GRADE-CERQual analysis and summarized them into thematic statements. AEP inductively analyzed the data and grouped related barriers and enablers judged to have high levels of confidence together, regardless of which domain they belonged to, into overarching thematic statements. These statements were reviewed by KB for completeness and accuracy. We limited this summary to the barriers and enablers with high confidence because of their importance to future intervention design and their likelihood of being a reasonable representation of the phenomenon of interest [33].

### Presentation of results

We have created separate summary of findings tables barriers and enablers. It is important to note that we have organized the summary of findings table using two levels of ordering: one based on the number of barriers or enablers identified at each TDF domain and one based on the amount of contributing data for each barrier or enabler. Thus, domains with the greatest number of barriers or enablers are at the top of the table, and within the domain, barriers or enablers with the greatest number of contributing studies are at the top of the domain. While we have grouped the findings in this way for ease of reading, we are not suggesting that these factors are indicative of the relative importance of any one domain, barrier or enabler. Rather, we think it is important to consider the findings together as described in the results where we present over-arching themes based on how the findings seem to relate to each other irrespective of their domain or number of contributing studies.

## Results

### Summary of included studies

The electronic search resulted in 2837 articles to be screened. Of these, 2759 were screened out following title and abstract review and 3 articles could not be retrieved, leaving 75 for full-text assessment. A total of 23 studies met our eligibility criteria. Reasons for exclusion included: wrong population (i.e., not a URTI or did not specify which respiratory conditions were included; *n* = 25), wrong phenomenon of interest (i.e., did not focus on FP antibiotic prescribing for URTIs; *n* = 7), wrong publication type (i.e., not a peer-reviewed published article; *n* = 3), wrong study design (i.e., not a qualitative study design, *n* = 3), articles with multiple reasons for exclusion were listed under the multiple factors category (*n* = 14). Thus, 23 studies were identified that reported on FPs’ barriers and enablers for guideline-based antibiotic prescribing for URTIs. See Fig. 1 – PRISMA flow diagram below for more details and additional file – 3 to see a list of excluded studies.

### Description of included studies

A description of the study characteristics can be found in Table 2 below. The 23 included studies were published between 1994–2024 and collected data from 16 countries (note one study collected data from two countries): the United Kingdom (*n* = 3), Australia (*n* = 2), Canada (*n* = 2), China (*n* = 2), Netherlands (*n* = 2), United States (*n* = 2), Sweden (*n* = 2), Albania (*n* = 1), Finland (*n* = 1), Germany (*n* = 1), India (*n* = 1), Ireland (*n* = 1), Latvia (*n* = 1), Lithuania (*n* = 1), Norway (*n* = 1), and Russia (*n* = 1). Most of the studies used interviews (*n* = 18) to collect data, while some used both focus groups (*n* = 3) and interviews (both *n* = 2), with most of the data collection happening in person (*n* = 13). Some studies collected data via the phone (*n* = 3), and some used both methods (*n* = 4). Three studies did not report their method of contact for their data collection. Most of the studies only collected data from FPs (*n* = 17); however, some included patients or other healthcare professionals (*n* = 6). A total of 516 FPs were included in this review, with some FP locums (*n* = 19) and some FP trainees (*n* = 38). Some studies examined URTIs collectively (*n* = 11), while others examined specific URTI conditions (*n* = 11). One study from India examined URTIs and diarrhea, and one study from the United Kingdom examined a collection of conditions for which antibiotics are not usually recommended (acute bronchitis, acute cough, acute otitis media, acute rhinosinusitis, acute sore throat, asthma exacerbations, and mild acute exacerbations of chronic obstructive pulmonary disease (COPD)). Data specific to diarrhea, COPD, and asthma were not extracted.

**Table 2:** Description of included studies

Author, Year, Country	Aim	Included condition/s	FPs (n)	Data Collection Method
Borek et al., 2022, United Kingdom [39]	To identify how locums’ antibiotic prescribing compares with other general practice prescribers, and how they perceive their role in antibiotic prescribing and AMS	Acute: bronchitis, cough, otitis media, rhinosinusitis, sore throat; exacerbations of asthma or COPD*	Locum FPs (19)	Interviews
Butler et al., 1998, United Kingdom [40]	To better understand reasons for antibiotics being prescribed for sore throats despite well-known evidence that they are generally of little help	Sore throats	FPs (21)	Interviews In-person
Dallas et al., 2014, Australia [41]	To explore the attitudes of trainees in general practice towards antibiotic use and resistance, and the perceived influences on their prescribing	URTIs (including acute bronchitis)	FP trainees (17)	1 Focus group & interviews Phone & in-person
Damoiseaux et al., 1999, Netherlands [42]	To explore the reasons, other than those stated in the guidelines of the Dutch College of FPs, for prescribing antibiotics for acute otitis media	Acute otitis media	FPs (22)	Interviews Mode not reported
de Bock et al., 1994, Netherlands [43]	To describe a qualitative analysis of a decision problem of acute maxillary sinusitis in general practice	Acute maxillary sinusitis	FPs (8)	Interviews In-person
Dempsey et al., 2014, United States [44]	To understand contemporary reasons for antibiotic prescribing for acute bronchitis in the United States	Acute bronchitis	FPs (12)	Interviews Phone
Fletcher-Lartey et al., 2016, Australia [45]	To explore the management of URTI and antibiotic prescribing in general practice in Australia	URTIs	FPs (32)	Interviews Phone & in-person
Hedin et al., 2014, Sweden [46]	To explore how a group of Swedish general practitioners (FPs) manage patients with a sore throat in relation to current guidelines as expressed in interviews	Sore throats	FPs (25)	Interviews In-person
Jaruseviciene et al., 2013, Lithuania & Russia [47]	To explore experiences of FPs in Lithuania and the Russian Federation with regard to antibiotic prescription for upper respiratory tract infections	URTIs	FPs (61)	5 Focus groups In-person
Kaae et al., 2017, Albania [48]	To investigate the antibiotic knowledge, attitudes and behaviours of patients and healthcare professionals in the country	URTIs	FPs (4)	Interviews Not reported
Kadirhaz et al., 2024, China [49]	To investigate how primary healthcare physicians make decisions regarding antibiotic prescription for URTIs, and to identify key factors that influence this decision-making process	URTIs	FPs (13) FP trainees (9)	Interviews In-person
Kotwani et al., 2017, India [50]	To explore the prescribing practices, knowledge, and attitudes of primary care doctors and community pharmacists, regarding antibiotic use in acute URTIs and diarrhea in children to better understand causes of misuse and identify provider suggestions to change such behavior	URTIs and diarrhea	FPs (45)	Interviews & 2 Focus groups In-person
Kumar et al., 2003, United Kingdom [49]	To understand why general practitioners, prescribe antibiotics for some cases of sore throat and to explore the factors that influence their prescribing	Sore throats	FPs (40)	Interviews In-person
Moe et al., 2021, Canada [51]	To examine the factors influencing family medicine residents’ antibiotic prescribing behaviour for upper respiratory tract infections (URTI) using the Theoretical Domains Framework (TDF)	URTIs	FPs trainees (12)	Interviews Phone
O’Doherty et al., 2019, Ireland [50]	To investigate why FPs continue to prescribe antibiotics for ARTIs despite increasing knowledge of their poor efficacy and worsening antimicrobial resistance	URTIs	FPs (13)	Interviews In-person
Patel et al., 2020, United States [49]	To identify factors associated with high and low prescriber status for management of URTIs in primary care practice	URTIs	FPs (29)	Interviews In-person & phone
Rutkovska et al., 2022, Latvia [50]	To explore the diagnostic and treatment process of tonsillopharyngitis by general practitioners and to understand decisions regarding antibiotic prescribing and the factors that shape these practices	Tonsillopharyngitis	FPs (8)	Interviews In-person
Schubert et al., 2023, Germany [49]	To understand how FPs conceptualize acute bronchitis and its border-categories, the common cold and pneumonia, how they discriminate between these three and where and why they see an indication for an antibiotic	Acute bronchitis	FPs (12)	Interviews In-person
Shen et al., 2023, China [50]	To explore how clinical uncertainty influences antibiotic prescribing practices among township hospital physicians and village doctors in rural Shandong Province, China	URTIs	FPs (30) Village doctors (6)	Interviews In-person
Simeoni et al., 2022, Canada [49]	To identify potentially modifiable determinants of antibiotic prescribing for patients presenting to primary care with upper respiratory tract infection symptoms	URTIs	FPs (20)	Interviews Phone
Thaulow et al., 2023, Norway [50]	To explore the clinical decision-making process and reasons for treatment with antibiotics for acute sinusitis among Norwegian general practitioners	Acute sinusitis	FPs (25)	5 Focus groups In-person
Tystrup et al., 2020 Sweden [49]	To explore FPs’ stated management of patients with cough, earache, and sore throat and their adherence to guidelines and to explore how these relate to the specific characteristics of each guideline	Cough, earache/infections and sore throats	FPs (29)	Interviews Not reported
Varonen et al., 2004, Finland [50]	To study the views of patients and physicians on the management of suspected acute maxillary sinusitis and on suggested changes in practice	Acute maxillary sinusitis	FPs (20)	8 Focus Groups In-Person

### Assessment of methodological rigour

**Fig. 2:**
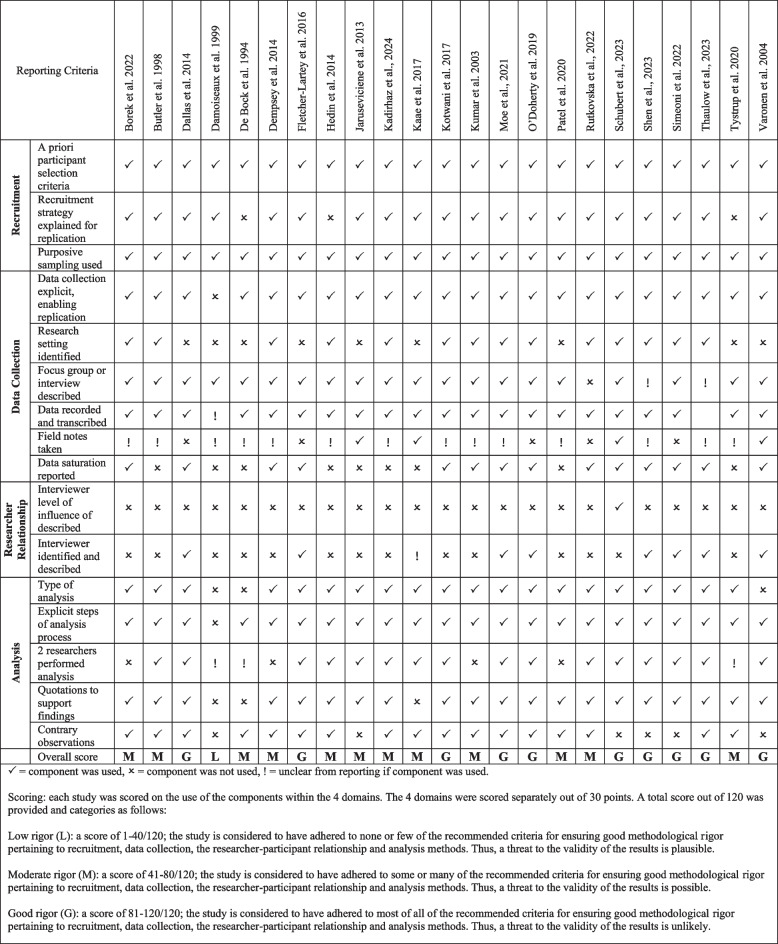
Methodological rigor assessment of included studies

Ten of the included studies were found to have good rigour [41, 45, 50–57], twelve of the were found to have moderate rigour [39, 40, 43, 44, 46–49, 58–61], and only one study had low rigor [42]. Most studies provided sufficient details on the qualitative approach, design, and recruitment strategies. However, researcher-participant relationship, ethics, and data collection methods were not reported well by the included studies. For example, only one included study provided sufficient information on the interviewer's level of influence, and only seven studies adequately described and identified the study's interviewer. Furthermore, only four studies reported taking field notes, and nine studies clearly reported the research setting. See Fig. 2 below for the full methodological rigour assessment and Additional file 4 for the complete assessment of reporting criteria according to the guidance from CASP and COREQ.

### Barriers to following antibiotics guidelines for URTIs

We identified 61 barriers in 13 TDF domains; we were highly confident in 17 barriers across 8 TDF domains, moderately confident in 7 barriers across 4 domains, and, had either low or very low confidence in 29 barriers across 9 domains. The 36 findings ranging from very low to moderate confidence were downgraded due to methodological issues, limited amounts of supporting data, and largely superficial data. Additional file 5 presents a summary of findings table (with supporting quotes) for all findings identified within each domain. The remainder of the results will focus on 17 barriers that we have high confidence in that they are highly likely to represent barriers to following antibiotic prescribing guidelines globally and should be addressed in future interventions. All included studies (*n* = 21) contributed data to at least one of the 17 barriers. Table 3 presents the summary of findings for these 17 barriers.

**Table 3:** GRADE CER-Qual summary of findings for barriers (high confidence only). PICO: What do family physicians believe are the barriers to not prescribing antibiotics for uncomplicated URTIs?

Themes (phrased as belief statements) that we judged to have a high level of confidence. i.e. we judged each of these findings to have no/very minor methodological concerns, to have coherent supporting data, to have adequate data richness and/or quantity, and to be highly relevant to our PICO question	studies # (sample)	Countries Represented	Year Range
Beliefs about consequences (*n* = 5 barriers)			
Prescribing antibiotics for URTIs helps to avoid missing complications or something significant [39, 41–45, 47, 48, 50–56, 58–60].	18(408)	AL, AU, CA, CN, DE, FI, IE, IN, LT, NL, RS, UK, US	1994–2024
Prescribing antibiotics for URTIs does not impact antibiotic resistance and can have benefits for patient care [40, 41, 43–45, 52, 54, 59, 61].	9(195)	AU, DE, IN, LV, NL, UK, US	1994–2023
Prescribe to not risk or preserve their doctor-patient relationships [40, 45, 53, 54, 56, 61].	6(106)	AU, CA, DE, IE, LT, UK	1998–2023
Prescribe antibiotics for URTIs to protect their business [40, 45, 52, 55, 56].	5(148)	AU, CA, CN, IN, UK	1998–2023
It takes too long to explain why antibiotics are not prescribed for URTIs [40, 41, 59].	3(78)	AU, UK	1998–2014
Social Influences (*n* = 4)			
FPs prescribe antibiotics for URTIs to meet patient expectations or demand (real or perceived) for antibiotics or for patient satisfaction [39–46, 48, 50, 52–57, 59–61].	21(436)	AL, AU, CA, CN, DE, FI, IE, IN, LT, NL, NO, SE, UK, US	1994–2024
Lack of patient relationship, prior knowledge of the patient, or patient’s culture/history can increase prescribing [39, 40, 44, 45, 54–57, 59, 60].	10(240)	AU, CA, CN, DE, NO, UK, US	1998–2023
Influenced negatively by or lacking support from colleagues, supervisors, or clinic to not prescribe antibiotics unnecessarily for URTIs [39, 41, 44, 46, 51, 55, 56, 58]	8(157)	AU, CA, CN, UK, US	2014–2024
FPs report that patients do not understand that antibiotics will not work, they do not have the capacity to understand, or not perceived as capable of taking care of themselves or their kids [40, 44, 45, 53, 59].	5(118)	AU, IE, UK, US	1998–2019
Environmental Context and Resources (*n* = 3)			
Time pressure, workload, or day of the week can influence FPs to prescribe antibiotics for URTIs [39–41, 45, 46, 51–54, 56, 59–61].	13(293)	AU, CA, DE, IE, IN, LV, NL, SE, UK, US	1998–2023
Lack of access to other healthcare or resources leads to more defensive prescribing [45, 48, 49, 51, 52, 54, 55, 58, 61].	9(194)	AU, AL, CA, CN, DE, IN, LV, SE	2016–2024
Workplace culture, environment, or location can influence FPs to prescribe antibiotics for URTIs [39, 52, 53, 56].	4(97)	CA, IE, IN, UK	2017–2022
Memory Attention and Decision Processes (*n* = 1)			
Uncertainty when diagnosing URTIs can lead to inappropriate antibiotic prescriptions for URTIs [40, 41, 44–46, 50, 51, 51, 53–56, 58].	12(236)	AU, CA, CN, DE, FI, IE, SE, UK, US	1998–2024
Knowledge(*n* = 1)			
Lack of knowledge or misinformation on antibiotics (e.g., when to prescribe antibiotics, how they work) and antibiotic resistance may lead to unnecessary antibiotic prescribing for URTIs [39–41, 44, 48, 51, 52, 58, 59].	9(193)	AL, AU, CA, CN, IN, UK, US	1998–2024
Beliefs About Capabilities (*n* = 1)			
Lack of confidence in their ability and/or knowledge to diagnose or manage URTIs correctly can lead to unnecessary antibiotic prescribing [41, 45, 52, 54, 56, 59, 60].	8(207)	AU, CA, DE, IN, UK, US	2014–2024
Social Professional Role and Identity (*n* = 1)			
FPs think prioritizing patient care is their priority, not antibiotic resistance or guideline-based care which can lead to unnecessary antibiotic prescribing [40, 46, 49, 56, 57, 59, 61].	7(168)	CA, NO, SE, UK	1998–2023
Emotion (*n* = 1)			
Fatigue or lack of enthusiasm can influence FPs to prescribe antibiotics unnecessarily for URTIs [40, 41, 54, 56, 61].	5(78)	AU, CA, DE, UK	1998–2023

This table is organized at two levels: first by the domains that had the most belief statements, such that the domain with the most belief statements is listed first. Second, within each domain, the belief statements are organized by the number of supporting studies, such that the belief statement with the most support studies is listed first. We are not making judgments about the relative importance of each of these belief statements

### Overarching synthesis of barriers (across domains) with high confidence

Three key themes were identified from the overarching synthesis across TDF domains of 17 barriers with high confidence, presented in no particular order of significance as we judge each of these areas to be worthy of consideration for intervention planning. See Additional File 4 for supporting quotes.

#### Physician fatigue and poor support for guideline-adherent prescribing

Physicians working in busy clinics and under the strain of time pressures (*domain: Environmental context and resources*) are influenced to prescribe antibiotics unnecessarily. They report feeling too tired or lacking enthusiasm (*domain: Emotion*) to follow prescribing guidelines. Physicians believe that patients do not understand explanations about when and why antibiotics are indicated (*domain: Social influences*) and that it takes too long to explain why they should not be prescribed in a given circumstance (*domain: Beliefs about consequences*). Physicians also reported that non-adherent prescribing behavior can also be impacted by a workplace culture that doesn’t value antibiotic stewardship (*domain: Environmental context and resources*) and/or a lack of support from colleagues and supervisors to follow prescribing guidelines (*domain: Social influences*).

#### Perceptions about patient demand

Physicians reported prescribing unindicated antibiotics to meet or satisfy patient treatment expectations (*domain: Social influences*). They believe that in doing so, they will preserve their therapeutic relationship with patients and simultaneously protect their business (*domain: Beliefs about consequences*). In cases where physicians don’t know their patients well or don’t have an established therapeutic relationship (e.g., a walk-in patient), they report feeling that their advice about antibiotics is not wanted and will rely on patients’ own understanding of their need for antibiotics leading to inappropriate prescribing (*domain: Social influences*).

#### Poor physician understanding of antibiotics, antibiotic resistance, diagnostic uncertainty, and their role in patient care

Some physicians lack understanding of or are misinformed about how antibiotics work, their utility for treating URTIs, and antibiotic resistance (*domain: Knowledge*). They may also struggle with diagnostic certainty related to viral versus bacterial URTIs (*domain: Memory, attention, and decision processes*) and lack confidence to diagnose and manage URTIs without prescribing antibiotics (*domain: Beliefs about capabilities*). Physicians may also prescribe defensively – to avoid missing a serious illness that does require antibiotics (*domain*: *Beliefs about consequences*). This defensive prescribing is worsened by reported poor access to other healthcare resources to help with definitive diagnosis (*domain: Environmental context and resources*). Finally, some physicians believe their role is to focus on patient care as opposed to antibiotic stewardship, failing to discern an overlap between the two (*domain: Social professional role and identity*). Some physicians believe that prescribing antibiotics could potentially benefit their patients without the risk of antibiotic resistance (*domain: Beliefs about consequences*).

### Enablers to following antibiotics guidelines for URTIs

We identified 40 enablers in 13 domains; we were highly confident in 10 enablers across 8 TDF domains, moderately confident in 12 enablers across 7 TDF domains, and had low or very low confidence in 18 enablers across 10 TDF domains. The 30 findings ranging from very low to moderate were downgraded due to methodological issues, limited amounts of supporting data, and largely superficial data. Additional file 4 presents a summary of findings table (with supporting quotes). Table 4 presents the summary of findings for the 10 enablers that were judged to have high confidence.

**Table 4:** GRADE CER-Qual summary of findings for enablers (high confidence only). PICO: What do family physicians believe are the enablers to not prescribing antibiotics for uncomplicated URTIs?

Themes (phrased as belief statements) that we judged to have a high level of confidence. i.e. we judged each of these findings to have no/very minor methodological concerns, to have coherent supporting data, to have adequate data richness and/or quantity, and to be highly relevant to our PICO question	studies # (sample)	Countries Represented	Year Range
Behavioural regulation (*n* = 2 enablers)			
Patient education [39, 40, 44, 45, 51, 56, 59, 60]	8(185)	AU, CA, UK, US	1998–2022
Delayed prescribing [40, 45, 53, 54, 56, 59, 61]	7(146)	AU, CA, DE, IE, LV, UK	1998–2023
Beliefs about consequences (*n* = 2)			
Overprescribing antibiotics has numerous negative consequences for URTI care and antibiotic resistance [40, 41, 43, 45, 48, 51]	6(94)	AL, AU, CA, NL, UK	1998–2021
Following guidelines protects FPs from medicolegal issues and grounds management decisions in evidence-based medicine. [41, 47, 61]	3(76)	AU, LT, LV, RU	2013–2023
Social Influences (*n* = 1)			
FPs noted that patients don't necessarily want antibiotics or expect them, and/or the demand for them has decreased or this dissatisfaction didn't bother them [40, 44, 46, 48, 58–60]	7(153)	AL, CN, SE, UK, US	1998–2024
Social and professional role and identity (*n* = 1)			
FPs think it is their responsibility to not overprescribe antibiotics for URTIs [39, 41, 44, 51, 56, 59]	6(120)	AU, CA, UK, US	2003–2022
Beliefs about capabilities (*n* = 1)			
Confidence in their abilities and knowledge helped FPs manage URTIs without prescribing antibiotics [41, 51, 56–58, 61]	6(104)	AU, CA, CN, LV, NO	2014–2024
Knowledge (*n* = 1)			
Knowledge of antibiotics and antibiotic resistance can lead to less antibiotic prescribing for URTIs [39, 40, 48, 53, 59]	5(97)	AL, IE, NO, UK	1998–2023
Goal (*n* = 1)			
Promote antibiotic stewardship and follow best practices [40, 51, 59, 60]	4(102)	CA, UK, US	1998–2021
Environmental context and resources (*n* = 1)			
Having educational resources for FPs or patients has helped FPs reduce prescribing [41, 44, 56]	3(49)	AU, CA, US	2014–2022

This table is organized at two levels: first by the domains that had the most belief statements, such that the domain with the most belief statements is listed first. Second, within each domain, the belief statements are organized by the number of supporting studies, such that the belief statement with the most support studies is listed first. We are not making judgments about the relative importance of each of these belief statements

### Overarching synthesis of enablers (across domains) with high confidence

Two key themes were identified from the overarching synthesis across TDF domains of 10 enablers with high confidence, presented in no particular order of significance as we judge each of these areas to be worthy of consideration for intervention design. See additional file 4 for supporting quotes.

#### Understanding antibiotic and antibiotic resistance and valuing evidence-based prescribing

Physicians who have a good understanding of the role of antibiotics in patient care and evidence of the role of overprescribing in antibiotic resistance are more likely to follow prescribing guidelines for URTIs (*domain: Knowledge*). Confidence in their abilities and knowledge about this issue also helps them to manage URTIs without antibiotics (*domain: Beliefs about capabilities*). Relatedly, some physicians feel it is a part of their role to avoid overprescription of antibiotics (*domain: Social professional role and identity*) and have goals related to promoting antibiotic stewardship and following best practices regarding prescribing (*domain: Goals*). They believe that following guidelines grounds their treatment in evidence-based medicine, thereby protecting them from medicolegal issues (*domain: Beliefs about consequences*).

#### Targeted strategies and tools focused on both physician and patient behavior

Physicians believe that using delayed prescriptions for their patients helps to avoid overprescribing (*domain: Behavioral regulation*). Physicians reported that their patients come to them with URTIs for diagnosis and reassurance that they don’t have a serious illness (*domain: Social influences*). To avoid overprescribing, physicians also reported that patient-facing education resources indicating when and why antibiotics should and shouldn’t be prescribed can help (*domain: Environmental context and resources; domain: Behavioral regulation*).

## Discussion

### Summary of findings

This review identified 23 qualitative studies with moderate to high methodological rigour that assessed the barriers and enablers to following antibiotic prescribing guidelines for URTIs and related conditions among 516 FPs in primary care. Our review provides an up-to-date synthesis (including nine new studies since the last relevant review) and provides a comprehensive TDF-based analysis and GRADE CERQual synthesis which can be used to develop theory-informed behaviour change interventions to improve antibiotic prescribing. We found 17 barriers and 10 enablers with high confidence, meaning that those barriers and enablers are likely to be a reasonable representation of the true barriers and enablers for FP adherence to antibiotic guidelines for URTIs. Overall, barriers to following antibiotics prescribing guidelines centred around (1) poor physician understanding of antibiotic resistance and antibiotics and their role in patient care, (2) lack of support for guideline-based prescribing and physician fatigue, and (3) perception of patient demand for antibiotics and the doctor-patient relationship. While enablers were not as commonly reported in the literature as barriers, we did find that (1) knowledge of the impact of antibiotic use on antibiotic resistance and prioritizing evidence-based care, and (2) antibiotic reduction strategies and tools for both physician and patient behaviour are factors that would support the appropriate antibiotic prescribing.

### Comparison with the literature

Several barriers and enablers identified in our review are consistent with those of other health behaviours that have been analyzed with the TDF [34, 62, 63]. Not surprisingly, and consistent with the literature, the barriers relating to patient influence, fear of missing something important, lack of resources, workload, and time pressure were among the most frequently reported. For example, similar to our finding that FP prescribing is influenced by perceived patient pressure or expectations (*domain: Social influences*), we found evidence that physicians report prescribing other medications due to patient pressure (e.g., benzodiazepines) despite their lack of efficacy in older adults [34, 63]. Additionally, our finding that FPs continue to prescribe antibiotics to avoid missing something important is also found in other health areas where a conclusive diagnosis cannot be made within the consultation [34]. We also found that lack of access to health resources was reported to lead to more defensive healthcare practices (i.e., prescribing antibiotics when they otherwise would not if they had access to diagnostic tools or testing facilities) which was consistent with the findings from Hall et al. who found that if FPs thought that there could be long wait times for imaging, they might order it early in case they needed it later [34]. Furthermore, time pressures was also a barrier to following guideline-based care for low back pain, COPD or asthma, and benzodiazepine use [34, 62, 63].

Knowledge about antibiotic prescribing also emerged as a barrier in our review, despite ongoing efforts to promote antibiotic stewardship in recent years. This finding is also supported in the literature. For example, two qualitative systematic reviews of physician antibiotic prescribing behaviour found that physicians have misconceptions or lack knowledge about antibiotic prescribing and the role overprescription of antibiotics plays in antibiotic resistance [64, 65]. Other studies that have assessed physician [66–69] or FP [70–73] antibiotic and antibiotic resistance knowledge via surveys have also found similar gaps in knowledge. Notably, knowledge about antibiotics and antibiotic resistance was found to be an enabler in our review, indicating that it is an important part of the process of changing behaviour. Thus, the fact that knowledge may still be lacking for FPs in numerous countries should not be taken for granted when planning interventions. Because attention to overprescribing and antibiotic resistance has increased considerably in recent years, we considered the possibility that clinician knowledge is no longer a factor affecting antibiotic prescribing; however, this issue was identified in a paper published as recently as 2023, suggesting knowledge is still important to consider. As described above, we know from our data that knowledge is only one part of a complex issue, and interventions will need to address more than knowledge to be successful.

### Implications for practice and research

The biggest implication of our study is the impact our results could have on the design and efficacy of future interventions for improving antibiotic prescribing for URTIs. The barriers and enablers we determined to have high confidence (i.e., more likely to be true barriers and enablers for FPs in primary care) should be used in future intervention design and development to improve intervention efficacy. As with any implementation intervention, it is important to consider the context/system in which it is taking place when confirming influencing factors. For example, local policies or interventions that are already in place or have been implemented in previous years may influence which barriers to target in intervention development. Thus, while we suggest the barriers/enablers in this review are a good place to start for designing interventions, we would also recommend a pre and/or parallel assessment of contextual factors to help refine or broaden intervention targets.

Several studies have designed and tested interventions to help reduce inappropriate antibiotic prescribing for URTIs [17, 18, 74]. These interventions generally contain one or more of the following components: communication training (e.g., communication strategies such as shared decision-making), clinical strategies (e.g., any strategy to improve care or diagnostic accuracy like delayed prescribing or clinical prediction rules), educational interventions (e.g., education on healthcare topics for clinicians, patients or both), point-of-care testing (e.g., a collection of tests that help clinicians diagnose URTIs), and system-level strategies (e.g., any strategy delivered at a system level such as audit and feedback or electronic reminders). Some of the interventions from the literature use strategies such as providing education on a topic that would target the barriers we identified under the TDF domain of *Knowledge* and clinical and system-level strategies of reminders which may help address the barriers we identified under the *Environmental context and resources* domain [17, 18]. However, many of the intervention strategies used in the literature to change prescribing behaviour do not address several of the barriers and enablers we found to have high-quality support. For example, some strategies used in the literature targeted domains that our results indicated to be not as important to address such as using communication training which would target barriers under the *Skills* domain. Furthermore, none of the strategies used in the literature directly address barriers under the domains of *Social influences*, *Beliefs about capabilities,* or *Beliefs about consequences* [17, 18]. Thus, this gap in addressing the underlying mechanisms of antibiotic overprescribing for URTIs may explain why these interventions are ineffective in changing behaviour.

Interventions can be designed to target and overcome TDF-identified barriers using the behaviour change technique (BCT) taxonomy. The BCT Taxonomy is a list of 93 behaviour change techniques developed in conjunction with the TDF to help design theory-informed interventions [22, 75–77]. Michie provides guidance on selecting the most appropriate BCT to target an identified barrier or enabler based on the TDF domain to which it was coded [75]. Below is an example of the tool being applied for the barrier regarding FP’s lack of confidence in their ability or knowledge in managing URTIs following URTI guidelines. This barrier falls under the domain of *Beliefs about capabilities*. According to Cane et al., the BCTs that target the barriers relating to beliefs about one’s capabilities are *verbal persuasion to boost self-efficacy* and *focus on past success* (see Table 5 below for definitions). Thus, interventions incorporating one of these BCTs are more likely to be effective in addressing the barrier related to FPs’ lack of confidence in their ability or knowledge in managing URTIs following URTI guidelines than previously implemented interventions [75, 78].

**Table 5:** Example of using the behaviour change techniques taxonomy to identify appropriate intervention strategies

Domain	Barrier	BCT options and definition
Beliefs about capabilities	Lack of confidence in their ability or knowledge to differentiate viral URTIs from bacterial infections can lead to unnecessary antibiotic prescribing	Verbal persuasion to boost self-efficacy: Provide arguments to counter the rationale for self-doubt (e.g., receiving positive verbal feedback or encouragement on prescribing behaviour) Focus on past Success: Document prior success and the lack of adverse consequences. (e.g., using scales to rate goal progress and self-efficacy related to guideline adherence)

While it is recommended that future interventions address the barriers and enablers that were determined to have high confidence, we also identified several barriers and enablers with moderate confidence that could influence practice behaviour (see additional file 4). These barriers and enablers were downgraded due to a low number of supporting studies or their relevance to our review as they included some conditions outside the scope of our study. More research is needed to confirm the validity of barriers and enablers with moderate confidence as it is possible that they might play a significant role in FPs’ antibiotic prescribing for URTIs, and therefore, may also need to be addressed in future intervention design. We would suggest that future studies assessing barriers/enablers or quality improvement initiatives assessing contextual issues consider including specific questions about these issues as well to confirm if they do influence behavior.

### Strengths

We published our protocol following guidelines from the PRISMA statement and the JBI Manual for Evidence Synthesis chapter on Systematic Reviews of Qualitative Evidence [29, 30]. In particular, we used a well-defined question following the recommended PICOS approach which allows our findings to be easily generalized and provide a more usable synthesis for those wishing to understand and change antibiotic prescribing for URTIs in primary care. Additionally, our search followed the PRESS guidelines for a comprehensive search strategy and all included studies were assessed for methodological rigour and reporting quality using the CASP and COREQ [31, 35, 36]. We adhered to the latest guidance on using the GRADE-CERQual approach to provide an indication of confidence in each review finding [38]. Lastly, we have followed reporting criteria from the PRISMA statement (See additional file 6) [33, 79].

### Limitations

While all efforts were made to keep the methodology for this review as rigorous as possible, there were some limitations. Due to resource restrictions, only one researcher was able to complete the TDF coding and the GRADE-CERqual analysis. However, a TDF expert did review all the data for accuracy and appropriateness. Additionally, the inclusion and exclusion screening criteria may have been too restrictive as it was often difficult to determine which infections were analyzed due to poor reporting. Thus, studies were excluded if the included conditions were not explicitly reported, which could have excluded relevant data. Similarly, we did not find any studies that discussed the COVID-19 pandemic and met our inclusion criteria (i.e., antibiotic prescribing for UTRIs), which may have influenced how physicians prescribe now and could be a relevant factor to consider for future intervention design. Furthermore, while studies from 16 different countries were included, much of our data comes from developed countries and we did not have any representation from Africa or Latin America. Thus, our findings may be limited in their generalizability to some settings or contexts.

## Conclusion

The overprescription of antibiotics for URTIs and URTI guideline adherence remains a significant problem in primary care globally. This review has found a high level of confidence in 17 barriers across 8 TDF domains and 10 enablers across 8 TDF domains that help us understand FP antibiotic prescribing behaviour for URTIs. The barriers we identified were largely related to misconceptions about how antibiotics work and antibiotic resistance, physician fatigue and lack of support, and perceived patient demand, whereas the enablers were related to understanding antibiotic resistance, valuing evidence-based prescribing, and target strategies for FPs and patients. To reduce the overprescription of antibiotics for URTIs and improve URTI guideline adherence, future interventions need to utilize behaviour change strategies that address these barriers and enablers. Additionally, there were several other barriers and enablers identified from this review but due to insufficient or low-quality data, we were less confident in their contribution to FP’s URTI antibiotic prescribing behaviour. While the barriers and enablers with a high level of confidence were consistent across multiple settings and countries, it is still necessary to consider any contextual factors when designing an intervention as they may influence which barriers should be targeted.

## Supplementary Information

Supplementary Material 1.

Supplementary Material 2.

Supplementary Material 3.

Supplementary Material 4.

Supplementary Material 5.

Supplementary Material 6.

## Data Availability

The datasets used and/or analysed during the current study are available from the corresponding author on reasonable request.

## Abbreviations

BCT/s: Behaviour change technique/s
CASP: Critical Appraisal Skills Program
COREQ: Consolidated Criteria for Reporting Qualitative Research
FPs: Family Physicians
GRADE-CERQual: Grading of Recommendations Assessment, Development, and Evaluation - Confidence in the Evidence from Reviews of Qualitative research
PRISMA: Preferred Reporting Items for Systematic Reviews and Meta-Analysis
PRISMA-P: Preferred Reporting Items for Systematic Reviews and Meta-Analysis Protocols
PRESS: Peer Review of Electronic Search Strategies
TDF: Theoretical domains framework
URTI/s: Upper respiratory tract infection/s
