# Supplementary material for: Barriers and enablers to using evidence-based antibiotic prescription guidelines in primary care: a qualitative systematic review and synthesis using the theoretical domains framework
Source: Implement Sci Commun. 2026 Feb 16;7:52. doi: 10.1186/s43058-025-00806-w (PMC13032215; doi:10.1186/s43058-025-00806-w)
Supplement: Supplementary file 1 — Supplementary Material 1. [file 43058_2025_806_MOESM1_ESM.docx]

Additional File 1 – Search strategy

**Database: Ovid MEDLINE(R) ALL**

1. Respiratory Tract Infections/
2. (respiratory adj2 infect*).tw,kf.
3. (urti or urtis).tw,kf.
4. exp Otitis Media/
5. (otitis or earache* or ear ache* or (ear adj1 infect*) or (ear adj1 inflam*)).tw,kf.
6. exp Pharyngitis/
7. (pharyngitis or nasopharyngitis or rhinopharyngitis or tonsillopharyngitis or tonsillitis or peritonsillar abscess* or retropharyngeal abscess* or sore throat*).tw,kf.
8. exp Sinusitis/
9. (sinusitis or rhinosinusitis).tw,kf.
10. exp Bronchitis/
11. (bronchitis or bronchiolitis).tw,kf.
12. Common Cold/
13. common cold*.tw,kf.
14. or/1-13
15. Anti-Bacterial Agents/
16. (antibiotic* or anti biotic* or antibacterial* or anti bacterial* or antimicrobial* or anti microbial*).tw,kf.
17. or/15-16
18. Primary Health Care/ or Physicians, Primary Care/ or exp General Practice/ or General Practitioners/ or Physicians, Family/
19. (primary care or primary health care or primary healthcare or general practi* or general physician* or GP or GPs or family practi* or family physician* or family doctor* or family medic*).tw,kf.
20. or/18-19
21. Attitude/ or "Attitude of Health Personnel"/ or Motivation/ or Intention/
22. (barrier* or obstacle* or challeng* or hinder* or hindrance* or disincentiv* or facilitat* or enabl* or incentiv* or attitude* or opinion* or perspective* or perception* or perceive* or view* or belie* or experience* or knowledge or understanding or motivat* or (factor* adj2 (influenc* or impact* or affect*))).tw,kf.
23. Qualitative Research/
24. Focus Groups/
25. Interview/ or "Interviews as Topic"/
26. Grounded Theory/
27. (qualitative or interview* or focus group* or ((thematic* or comparative*) adj1 analy*) or lived experience* or mixed method* or grounded theory or phenomenolog* or ethnograph*).tw,kf.
28. or/21-27
29. 14 and 17 and 20 and 28

**Embase (via Embase.com)**

|  | **Embase Query** |
| --- | --- |
| #1 | 'respiratory tract infection'/de OR 'upper respiratory tract infection'/de OR 'viral upper respiratory tract infection'/de |
| #2 | (respiratory NEAR/2 infect*):ti,ab,kw |
| #3 | urti:ti,ab,kw OR urtis:ti,ab,kw |
| #4 | 'otitis media'/exp |
| #5 | otitis:ti,ab,kw OR earache*:ti,ab,kw OR 'ear ache*':ti,ab,kw OR ((ear NEAR/1 infect*):ti,ab,kw) OR ((ear NEAR/1 inflam*):ti,ab,kw) |
| #6 | 'pharyngitis'/exp |
| #7 | pharyngitis:ti,ab,kw OR nasopharyngitis:ti,ab,kw OR rhinopharyngitis:ti,ab,kw OR tonsillopharyngitis:ti,ab,kw OR tonsillitis:ti,ab,kw OR 'peritonsillar abscess*':ti,ab,kw OR 'retropharyngeal abscess*':ti,ab,kw OR 'sore throat*':ti,ab,kw |
| #8 | 'sinusitis'/exp |
| #9 | sinusitis:ti,ab,kw OR rhinosinusitis:ti,ab,kw |
| #10 | 'bronchitis'/exp |
| #11 | bronchitis:ti,ab,kw OR bronchiolitis:ti,ab,kw |
| #12 | 'common cold'/de |
| #13 | 'common cold*':ti,ab,kw |
| #14 | #1 OR #2 OR #3 OR #4 OR #5 OR #6 OR #7 OR #8 OR #9 OR #10 OR #11 OR #12 OR #13 |
| #15 | 'antiinfective agent'/de OR 'antibiotic agent'/de |
| #16 | antibiotic*:ti,ab,kw OR 'anti biotic*':ti,ab,kw OR antibacterial*:ti,ab,kw OR 'anti bacterial*':ti,ab,kw OR antimicrobial*:ti,ab,kw OR 'anti microbial*':ti,ab,kw |
| #17 | #15 OR #16 |
| #18 | 'primary health care'/exp OR 'general practice'/de OR 'general practitioner'/de |
| #19 | 'primary care':ti,ab,kw OR 'primary health care':ti,ab,kw OR 'primary healthcare':ti,ab,kw OR 'general practi*':ti,ab,kw OR 'general physician*':ti,ab,kw OR GP:ti,ab,kw OR GPs:ti,ab,kw OR 'family practi*':ti,ab,kw OR 'family physician*':ti,ab,kw OR 'family doctor*':ti,ab,kw OR 'family medic*':ti,ab,kw |
| #20 | #18 OR #19 |
| #21 | 'attitude'/de OR 'health personnel attitude'/de OR 'physician attitude'/de OR 'patient attitude'/de OR 'motivation'/exp |
| #22 | barrier*:ti,ab,kw OR obstacle*:ti,ab,kw OR challeng*:ti,ab,kw OR hinder*:ti,ab,kw OR hindrance*:ti,ab,kw OR disincentiv*:ti,ab,kw OR facilitat*:ti,ab,kw OR enabl*:ti,ab,kw OR incentiv*:ti,ab,kw OR attitude*:ti,ab,kw OR opinion*:ti,ab,kw OR perspective*:ti,ab,kw OR perception*:ti,ab,kw OR perceive*:ti,ab,kw OR view*:ti,ab,kw OR belie*:ti,ab,kw OR experience*:ti,ab,kw OR knowledge:ti,ab,kw OR understanding:ti,ab,kw OR motivat*:ti,ab,kw OR ((factor* NEAR/2 (influenc* OR impact* OR affect*)):ti,ab,kw) |
| #23 | 'qualitative research'/exp |
| #24 | 'focus group'/exp |
| #25 | 'interview'/exp |
| #26 | 'grounded theory'/de |
| #27 | 'thematic analysis'/de |
| #28 | qualitative:ti,ab,kw OR interview*:ti,ab,kw OR 'focus group*':ti,ab,kw OR (((thematic* OR comparative*) NEAR/1 analy*):ti,ab,kw) OR 'lived experience*':ti,ab,kw OR 'mixed method*':ti,ab,kw OR 'grounded theory':ti,ab,kw OR phenomenolog*:ti,ab,kw OR ethnograph*:ti,ab,kw |
| #29 | #21 OR #22 OR #23 OR #24 OR #25 OR #26 OR #27 OR #28 |
| #30 | #14 AND #17 AND #20 AND #29 |

**Cochrane Library (via Wiley)**

| **ID** | **Cochrane Library Query** |
| --- | --- |
| #1 | [mh ^"Respiratory Tract Infections"] |
| #2 | (respiratory NEAR/1 infect*):ti,ab,kw |
| #3 | (urti OR urtis):ti,ab,kw |
| #4 | [mh "Otitis Media"] |
| #5 | (otitis OR earache* OR (ear NEXT ache*) OR (ear NEXT infect*) OR (ear NEXT inflam*)):ti,ab,kw |
| #6 | [mh Pharyngitis] |
| #7 | (pharyngitis OR nasopharyngitis OR rhinopharyngitis OR tonsillopharyngitis OR tonsillitis OR (peritonsillar NEXT abscess*) OR (retropharyngeal NEXT abscess*) OR (sore NEXT throat*)):ti,ab,kw |
| #8 | [mh Sinusitis] |
| #9 | (sinusitis OR rhinosinusitis):ti,ab,kw |
| #10 | [mh Bronchitis] |
| #11 | (bronchitis OR bronchiolitis):ti,ab,kw |
| #12 | [mh ^"Common Cold"] |
| #13 | (common NEXT cold*):ti,ab,kw |
| #14 | #1 OR #2 OR #3 OR #4 OR #5 OR #6 OR #7 OR #8 OR #9 OR #10 OR #11 OR #12 OR #13 |
| #15 | [mh "Anti-Bacterial Agents"] |
| #16 | (antibiotic* OR (anti NEXT biotic*) OR antibacterial* OR (anti NEXT bacterial*) OR antimicrobial* OR (anti NEXT microbial*)):ti,ab,kw |
| #17 | #15 OR #16 |
| #18 | [mh ^"Primary Health Care"] OR [mh ^"Physicians, Primary Care"] OR [mh "General Practice"] OR [mh ^"General Practitioners"] OR [mh ^"Physicians, Family"] |
| #19 | ("primary care" OR "primary health care" OR "primary healthcare" OR (general NEXT practi*) OR (general NEXT physician*) OR GP OR GPs OR (family NEXT practi*) OR (family NEXT physician*) OR (family NEXT doctor*) OR (family NEXT medic*)):ti,ab,kw |
| #20 | #18 OR #19 |
| #21 | [mh ^Attitude] OR [mh ^"Attitude of Health Personnel"] OR [mh ^Motivation] OR [mh ^Intention] |
| #22 | (barrier* OR obstacle* OR challeng* OR hinder* OR hindrance* OR disincentiv* OR facilitat* OR enabl* OR incentiv* OR attitude* OR opinion* OR perspective* OR perception* OR perceive* OR view* OR belie* OR experience* OR knowledge OR understanding OR motivat* OR (factor* NEAR/1 (influenc* OR impact* OR affect*))):ti,ab,kw |
| #23 | [mh ^"Qualitative Research"] |
| #24 | [mh ^"Focus Groups"] |
| #25 | [mh ^Interview] OR [mh ^"Interviews as Topic"] |
| #26 | [mh ^"Grounded Theory"] |
| #27 | (qualitative OR interview* OR focus group* OR ((thematic* OR comparative*) NEAR/1 analy*) OR (lived NEXT experience*) OR (mixed NEXT method*) OR "grounded theory" OR phenomenolog* OR ethnograph*):ti,ab,kw |
| #28 | #21 OR #22 OR #23 OR #24 OR #25 OR #26 OR #27 |
| #29 | #14 AND #17 AND #20 AND #28 |

**APA PsycINFO (EBSCOhost)**

| **#** | **APA PsycINFO Query** |
| --- | --- |
| S1 | DE "Respiratory Tract Disorders" |
| S2 | TI (respiratory N1 infect*) OR AB (respiratory N1 infect*) OR KW (respiratory N1 infect*) |
| S3 | TI (urti OR urtis) OR AB (urti OR urtis) OR KW (urti OR urtis) |
| S4 | TI ((otitis OR earache* OR "ear ache*" OR (ear N0 infect*) OR (ear N0 inflam*)) OR AB ((otitis OR earache* OR "ear ache*" OR (ear N0 infect*) OR (ear N0 inflam*)) OR KW ((otitis OR earache* OR "ear ache*" OR (ear N0 infect*) OR (ear N0 inflam*)) |
| S5 | TI (pharyngitis OR nasopharyngitis OR rhinopharyngitis OR tonsillopharyngitis OR tonsillitis OR "peritonsillar abscess*" OR "retropharyngeal abscess*" OR "sore throat*") OR AB (pharyngitis OR nasopharyngitis OR rhinopharyngitis OR tonsillopharyngitis OR tonsillitis OR "peritonsillar abscess*" OR "retropharyngeal abscess*" OR "sore throat*") OR KW (pharyngitis OR nasopharyngitis OR rhinopharyngitis OR tonsillopharyngitis OR tonsillitis OR "peritonsillar abscess*" OR "retropharyngeal abscess*" OR "sore throat*") |
| S6 | TI (sinusitis OR rhinosinusitis) OR AB (sinusitis OR rhinosinusitis) OR KW (sinusitis OR rhinosinusitis) |
| S7 | TI (bronchitis OR bronchiolitis) OR AB (bronchitis OR bronchiolitis) OR KW (bronchitis OR bronchiolitis) |
| S8 | TI ("common cold*") OR AB ("common cold*") OR KW ("common cold*") |
| S9 | S1 OR S2 OR S3 OR S4 OR S5 OR S6 OR S7 OR S8 |
| S10 | DE "Antibiotics" |
| S11 | TI (antibiotic* OR "anti biotic*" OR antibacterial* OR "anti bacterial*" OR antimicrobial* OR "anti microbial*") OR AB (antibiotic* OR "anti biotic*" OR antibacterial* OR "anti bacterial*" OR antimicrobial* OR "anti microbial*") OR AB (antibiotic* OR "anti biotic*" OR antibacterial* OR "anti bacterial*" OR antimicrobial* OR "anti microbial*") |
| S12 | S10 OR S11 |
| S13 | DE "Primary Health Care" OR DE "Family Medicine" OR DE "Family Physicians" OR DE "General Practitioners" |
| S14 | TI ("primary care" OR "primary health care" OR "primary healthcare" OR "general practi*" OR "general physician*" OR GP OR GPs OR "family practi*" OR "family physician*" OR "family doctor*" OR "family medic*") OR AB ("primary care" OR "primary health care" OR "primary healthcare" OR "general practi*" OR "general physician*" OR GP OR GPs OR "family practi*" OR "family physician*" OR "family doctor*" OR "family medic*") OR KW ("primary care" OR "primary health care" OR "primary healthcare" OR "general practi*" OR "general physician*" OR FP OR FPs OR "family practi*" OR "family physician*" OR "family doctor*" OR "family medic*") |
| S15 | S13 OR S14 |
| S16 | DE "Attitudes" OR DE "Health Attitudes" OR DE "Health Personnel Attitudes" OR DE "Motivation" OR DE "Intention" OR DE "Behavioral Intention" |
| S17 | TI (barrier* OR obstacle* OR challeng* OR hinder* OR hindrance* OR disincentiv* OR facilitat* OR enabl* OR incentiv* OR attitude* OR opinion* OR perspective* OR perception* OR perceive* OR view* OR belie* OR experience* OR knowledge OR understanding OR motivat* OR (factor* N1 (influenc* OR impact* OR affect*))) OR AB (barrier* OR obstacle* OR challeng* OR hinder* OR hindrance* OR disincentiv* OR facilitat* OR enabl* OR incentiv* OR attitude* OR opinion* OR perspective* OR perception* OR perceive* OR view* OR belie* OR experience* OR knowledge OR understanding OR motivat* OR (factor* N1 (influenc* OR impact* OR affect*))) OR KW (barrier* OR obstacle* OR challeng* OR hinder* OR hindrance* OR disincentiv* OR facilitat* OR enabl* OR incentiv* OR attitude* OR opinion* OR perspective* OR perception* OR perceive* OR view* OR belie* OR experience* OR knowledge OR understanding OR motivat* OR (factor* N1 (influenc* OR impact* OR affect*))) |
| S18 | DE "Qualitative Methods" |
| S19 | DE "Focus Group" OR DE "Focus Group Interview" |
| S20 | DE "Interviews" OR DE "Semi-Structured Interview" |
| S21 | DE "Grounded Theory" |
| S22 | DE "Interpretative Phenomenological Analysis" OR DE "Narrative Analysis" OR DE "Thematic Analysis" |
| S23 | TI (qualitative OR interview* OR "focus group*" OR ((thematic* OR comparative*) N1 analy*) OR "lived experience*" OR "mixed method*" OR "grounded theory" OR phenomenolog* OR ethnograph*) OR AB (qualitative OR interview* OR "focus group*" OR ((thematic* OR comparative*) N1 analy*) OR "lived experience*" OR "mixed method*" OR "grounded theory" OR phenomenolog* OR ethnograph*) OR KW (qualitative OR interview* OR "focus group*" OR ((thematic* OR comparative*) N1 analy*) OR "lived experience*" OR "mixed method*" OR "grounded theory" OR phenomenolog* OR ethnograph*) |
| S24 | S16 OR S17 OR S18 OR S19 OR S20 OR S21 OR S22 OR S23 |
| S25 | S9 AND S12 AND S15 AND S24 |

**CINAHL Plus with Full Text (EBSCOhost)**

| **#** | **CINAHL Query** |
| --- | --- |
| S01 | (MH "Respiratory Tract Infections") |
| S02 | TI (respiratory N1 infect*) OR AB (respiratory N1 infect*) |
| S03 | TI (urti OR urtis) OR AB (urti OR urtis) |
| S04 | (MH "Otitis Media+") |
| S05 | TI ((otitis OR earache* OR "ear ache*" OR (ear N0 infect*) OR (ear N0 inflam*)) OR AB ((otitis OR earache* OR "ear ache*" OR (ear N0 infect*) OR (ear N0 inflam*)) |
| S06 | (MH "Pharyngitis") |
| S07 | TI (pharyngitis OR nasopharyngitis OR rhinopharyngitis OR tonsillopharyngitis OR tonsillitis OR "peritonsillar abscess*" OR "retropharyngeal abscess*" OR "sore throat*") OR AB (pharyngitis OR nasopharyngitis OR rhinopharyngitis OR tonsillopharyngitis OR tonsillitis OR "peritonsillar abscess*" OR "retropharyngeal abscess*" OR "sore throat*") |
| S08 | (MH "Sinusitis+") |
| S09 | TI (sinusitis OR rhinosinusitis) OR AB (sinusitis OR rhinosinusitis) |
| S10 | (MH "Bronchitis+") |
| S11 | TI (bronchitis OR bronchiolitis) OR AB (bronchitis OR bronchiolitis) |
| S12 | (MH "Common Cold") |
| S13 | TI ("common cold*") OR AB ("common cold*") |
| S14 | S1 OR S2 OR S3 OR S4 OR S5 OR S6 OR S7 OR S8 OR S9 OR S10 OR S11 OR S12 OR S13 |
| S15 | (MH "Antibiotics") |
| S16 | TI (antibiotic* OR "anti biotic*" OR antibacterial* OR "anti bacterial*" OR antimicrobial* OR "anti microbial*") OR AB (antibiotic* OR "anti biotic*" OR antibacterial* OR "anti bacterial*" OR antimicrobial* OR "anti microbial*") |
| S17 | S15 OR S16 |
| S18 | (MH "Primary Health Care") OR (MH "Physicians, Family") OR (MH "Family Practice") |
| S19 | TI ("primary care" OR "primary health care" OR "primary healthcare" OR "general practi*" OR "general physician*" OR FP OR FPs OR "family practi*" OR "family physician*" OR "family doctor*" OR "family medic*") OR AB ("primary care" OR "primary health care" OR "primary healthcare" OR "general practi*" OR "general physician*" OR GP OR GPs OR "family practi*" OR "family physician*" OR "family doctor*" OR "family medic*") |
| S20 | S18 OR S19 |
| S21 | (MH "Attitude") OR (MH "Attitude of Health Personnel") OR (MH "Physician Attitudes") OR (MH "Attitude to Medical Treatment") OR (MH "Attitude to Health+") OR (MH "Patient Attitudes") OR (MH "Motivation") |
| S22 | TI (barrier* OR obstacle* OR challeng* OR hinder* OR hindrance* OR disincentiv* OR facilitat* OR enabl* OR incentiv* OR attitude* OR opinion* OR perspective* OR perception* OR perceive* OR view* OR belie* OR experience* OR knowledge OR understanding OR motivat* OR (factor* N1 (influenc* OR impact* OR affect*))) OR AB (barrier* OR obstacle* OR challeng* OR hinder* OR hindrance* OR disincentiv* OR facilitat* OR enabl* OR incentiv* OR attitude* OR opinion* OR perspective* OR perception* OR perceive* OR view* OR belie* OR experience* OR knowledge OR understanding OR motivat* OR (factor* N1 (influenc* OR impact* OR affect*))) |
| S23 | (MH "Qualitative Studies+") |
| S24 | (MH "Focus Groups") |
| S25 | (MH "Interviews+") OR (MH "Narratives") |
| S26 | TI (qualitative OR interview* OR "focus group*" OR ((thematic* OR comparative*) N1 analy*) OR "lived experience*" OR "mixed method*" OR "grounded theory" OR phenomenolog* OR ethnograph*) OR AB (qualitative OR interview* OR "focus group*" OR ((thematic* OR comparative*) N1 analy*) OR "lived experience*" OR "mixed method*" OR "grounded theory" OR phenomenolog* OR ethnograph*) |
| S27 | S21 OR S22 OR S23 OR S24 OR S25 OR S26 |
| S28 | S14 AND S17 AND S20 AND S27 |

**Web of Science Core Collection**

Editions:

- Science Citation Index Expanded (SCI-EXPANDED)--1900-present
- Social Sciences Citation Index (SSCI)--1956-present
- Arts & Humanities Citation Index (AHCI)--1975-present
- Emerging Sources Citation Index (ESCI)--2017-present

|  | **Web of Science Query** |
| --- | --- |
| 1 | TS=(respiratory NEAR/1 infect*) |
| 2 | TS=(urti OR urtis) |
| 3 | TS=(otitis OR earache* OR "ear ache*" OR (ear NEAR/0 infect*) OR (ear NEAR/0 inflam*)) |
| 4 | TS=(pharyngitis OR nasopharyngitis OR rhinopharyngitis OR tonsillopharyngitis OR tonsillitis OR "peritonsillar abscess*" OR "retropharyngeal abscess*" OR "sore throat*") |
| 5 | TS=(sinusitis OR rhinosinusitis) |
| 6 | TS=(bronchitis OR bronchiolitis) |
| 7 | TS=("common cold*") |
| 8 | #1 OR #2 OR #3 OR #4 OR #5 OR #6 OR #7 |
| 9 | TS=(antibiotic* OR "anti biotic*" OR antibacterial* OR "anti bacterial*" OR antimicrobial* OR "anti microbial*") |
| 10 | TS=("primary care" OR "primary health care" OR "primary healthcare" OR "general practi*" OR "general physician*" OR GP OR GPs OR "family practi*" OR "family physician*" OR "family doctor*" OR "family medic*") |
| 11 | TS=(barrier* OR obstacle* OR challeng* OR hinder* OR hindrance* OR disincentiv* OR facilitat* OR enabl* OR incentiv* OR attitude* OR opinion* OR perspective* OR perception* OR perceive* OR view* OR belie* OR experience* OR knowledge OR understanding OR motivat* OR (factor* NEAR/1 (influenc* OR impact* OR affect*))) |
| 12 | TS=(qualitative OR interview* OR "focus group*" OR ((thematic* OR comparative*) NEAR/0 analy*) OR "lived experience*" OR "mixed method*" OR "grounded theory" OR phenomenolog* OR ethnograph*) |
| 13 | #11 OR #12 |
| 14 | #8 AND #9 AND #10 AND #13 |
