# Supplementary material for: Barriers and enablers to using evidence-based antibiotic prescription guidelines in primary care: a qualitative systematic review and synthesis using the theoretical domains framework
Source: Implement Sci Commun. 2026 Feb 16;7:52. doi: 10.1186/s43058-025-00806-w (PMC13032215; doi:10.1186/s43058-025-00806-w)
Supplement: Supplementary file 2 — Supplementary Material 2. [file 43058_2025_806_MOESM2_ESM.docx]

**Additional File 2 – Additional GRADE CERQual rules**

1. Our protocol didn’t include a GRADE rule for how to make judgments about our confidence in review findings that were derived from a single study. However, we did come across this issue several times in our review and thus our team had discussions about how to make judgements particularly regarding the domains of adequacy and coherence. Our rule is as follows:

For review findings that were derived from a single study we would initially start the GRADE CERQual assessment at low confidence based on the likelihood of having serious concerns regarding insufficient data adequacy and an inability to fully assess coherence of the supporting data. Confidence in the review finding would be further downgraded if there were serious concerns regarding methodological rigour and/or relevance as per our existing GRADE CERQual rules for these domains. However, we considered there may be situations in which we would choose to upgrade the evidence for the domains of adequacy and coherence. The situations in which we may choose to upgrade the evidence are as follows:

- **Adequacy:** our moderate or serious concern about data adequacy for a review finding derived from a single study may be reduced to minor concern if both of the following *conditions* are present in the single study:
  1. If the data supporting the review finding is derived from a study that has a broad/diverse sample of family physicians including high and low prescribers of antibiotics and includes a range of gender, years of clinical experience that would be representative of clinical practice

AND

- 1. the data is sufficiently rich detailed enough to allow the review author to fully interpret the meaning and context of the finding in relation to prescribing antibiotics for URTIs when not indicated.

If both of these above conditions are present, we would no longer have serious concerns about the data adequacy and feel confident to upgrade the evidence for this domain.

- **Coherence**: in cases where we no longer had moderate or serious concerns about data adequacy
  1. **Ability to assess presence of contradictory / disconfirming cases** - If the data from the single study were obtained from an interview guide that was based on the theoretical domains framework which is the framework we are using for our analysis we may be able to better assess if contradictory findings were investigated. This is because a TDF based interview guide usually includes questions that ask participants about the presence of barriers and enablers at each of the 14 domains, thus allowing us to determine if contradictory findings were observed. Additionally, in TDF based interviews, if barriers or enablers are identified at a particular domain, further prompts are used to understand that barrier or enabler in more detail in relation the phenomenon of interest (i.e. prescribing antibiotics for uncomplicated URTIs), this should allow us to have more clarity regarding the supporting data and how well it fits the review finding from the study.
  2. **Variation in contextual assessment: If the single study assessed the phenomenon of interest in different contexts**.

1. Our protocol didn’t include a GRADE rule for how to make judgments about our confidence in review findings that were specific to a particular context. However, we did come across this issue a couple of times in our review and thus our team had discussions about how to make judgements particularly regarding the domain of relevance. Our rule is as follows:

- Our review question as outlined in the protocol: What are the barriers and enablers to prescribing antibiotics for uncomplicated URTIs in primary care settings as per evidence based recommendations in the Cold Standard? We further defined the context in our protocol to include all countries making our review question more relevant to answer barriers and enablers at a global level. However, as we moved through our review, we noticed that there were some barriers/enablers that may be more specific to certain contexts or countries and it would be unclear how these translated to other other settings. We decided to capture this issue within our GRADE Cer-qual assessment of the domain of relevance which is related to our review questions. Since the review question was global in nature we decided to consider downgrading review findings that were derived from studies from a single country or a particular context that was not global in nature and the type of review finding was unclear or unlikely to be present in other countries or other contexts. Below are two examples that we have come across and our rule for how to make judgements on the certainty in the relevance of these findings for our review question:
  1. **Country specific**: if the review finding was based on data from studies of a single country and the review finding was likely to be specific to that country we would have moderate or serious concerns that the review findings may not be relevant in other countries and thus we would downgrade the evidence here.
  2. **Primary care funding models**: if the review finding was in relation to a specific funding model and the review finding was likely to be specific to that funding model, we would have moderate or serious concerns that the review findings may not be relevant to other primary care funding models and thus we would downgrade the evidence here.
  3. **Single URTI Condition**: if the review finding was in relation to a specific URTI condition and the review finding was likely to be specific to that URTI condition, we would have moderate or serious concerns that the review findings may not be relevant to other URTI conditions and thus we would downgrade the evidence here.
