# Supplementary material for: Barriers and enablers to using evidence-based antibiotic prescription guidelines in primary care: a qualitative systematic review and synthesis using the theoretical domains framework
Source: Implement Sci Commun. 2026 Feb 16;7:52. doi: 10.1186/s43058-025-00806-w (PMC13032215; doi:10.1186/s43058-025-00806-w)
Supplement: Supplementary file 3 — Supplementary Material 3. [file 43058_2025_806_MOESM3_ESM.docx]

Additional File 3 - List of excluded studies

| Study | Reason for Exclusion |
| --- | --- |
| Altaye FW, Thupayagale-Tshweneagae G, Mfidi FH. Qualitative enquiry on factors affecting antibiotic prescribing at primary healthcare facilities in Addis Ababa, Ethiopia. Front Med (Lausanne). 2024 Apr 8;11:1308699. doi: 10.3389/fmed.2024.1308699. PMID: 38651064; PMCID: PMC11034364. | Wrong population |
| Andre, M., Gröndal, H., Strandberg, E. L., Brorsson, A., & Hedin, K. (2016). Uncertainty in clinical practice - an interview study with Swedish GPs on patients with sore throat. *BMC family practice*, *17*, 56. https://doi-org.qe2a-proxy.mun.ca/10.1186/s12875-016-0452-9 | Wrong phenomenon of interest |
| Ashdown, H. F., Räisänen, U., Wang, K., Ziebland, S., Harnden, A., & ARCHIE investigators* (2016). Prescribing antibiotics to 'at-risk' children with influenza-like illness in primary care: qualitative study. *BMJ open*, *6*(6), e011497. https://doi-org.qe2a-proxy.mun.ca/10.1136/bmjopen-2016-011497 | Wrong population |
| Barden, L. S., Dowell, S. F., Schwartz, B., & Lackey, C. (1998). Current attitudes regarding use of antimicrobial agents: results from physician's and parents' focus group discussions. *Clinical pediatrics*, *37*(11), 665–671. https://doi-org.qe2a-proxy.mun.ca/10.1177/000992289803701104 | Wrong population |
| Biezen, R., Brijnath, B., Grando, D., & Mazza, D. (2017). Management of respiratory tract infections in young children-A qualitative study of primary care providers' perspectives. *NPJ primary care respiratory medicine*, *27*(1), 15. https://doi-org.qe2a-proxy.mun.ca/10.1038/s41533-017-0018-x | Wrong population |
| Biezen, R., Grando, D., Mazza, D. et al. Dissonant views - GPs’ and parents’ perspectives on antibiotic prescribing for young children with respiratory tract infections. BMC Fam Pract 20, 46 (2019). https://doi-org.qe2a-proxy.mun.ca/10.1186/s12875-019-0936-5 | Wrong population |
| Bisgaard, L., Andersen, C. A., Jensen, M. S. A., Bjerrum, L., & Hansen, M. P. (2021). Danish GPs' Experiences When Managing Patients Presenting to General Practice with Symptoms of Acute Lower Respiratory Tract Infections: A Qualitative Study. *Antibiotics (Basel, Switzerland)*, *10*(6), 661. https://doi-org.qe2a-proxy.mun.ca/10.3390/antibiotics10060661 | Wrong population |
| Björnsdóttir, I., Kristinsson, K. G., & Hansen, E. H. (2010). Diagnosing infections: a qualitative view on prescription decisions in general practice over time. *Pharmacy world & science : PWS*, *32*(6), 805–814. https://doi-org.qe2a-proxy.mun.ca/10.1007/s11096-010-9441-6 | Multiple reasons |
| Boaitey, K. P., Hoffmann, T., Baillie, E., & Bakhit, M. (2023). Exploring general practitioners' perception of the value of natural history information and their awareness and use of guidelines' resources to support antibiotic prescribing for self-limiting infections: a qualitative study in Australian general practice. *Australian journal of primary health*, *29*(6), 558–565. https://doi-org.qe2a-proxy.mun.ca/10.1071/PY22258 | Multiple reasons |
| Bordado Sköld, M., Aabenhus, R., Guassora, A. D., & Mäkelä, M. (2017). Antibiotic treatment failure when consulting patients with respiratory tract infections in general practice. A qualitative study to explore Danish general practitioners' perspectives. *The European journal of general practice*, *23*(1), 120–127. https://doi-org.qe2a-proxy.mun.ca/10.1080/13814788.2017.1305105 | Wrong population |
| Borek, A.J., Campbell, A., Dent, E. *et al.* Development of an intervention to support the implementation of evidence-based strategies for optimising antibiotic prescribing in general practice. *Implement Sci Commun* **2**, 104 (2021). https://doi.org/10.1186/s43058-021-00209-7 | Wrong phenomenon of interest |
| Brookes-Howell, L., Hood, K., Cooper, L., Little, P., Verheij, T., Coenen, S., Godycki-Cwirko, M., Melbye, H., Borras-Santos, A., Worby, P., Jakobsen, K., Goossens, H., & Butler, C. C. (2012). Understanding variation in primary medical care: a nine-country qualitative study of clinicians' accounts of the non-clinical factors that shape antibiotic prescribing decisions for lower respiratory tract infection. *BMJ open*, *2*(4), e000796. https://doi-org.qe2a-proxy.mun.ca/10.1136/bmjopen-2011-000796 | Wrong population |
| Brookes-Howell, L.; Butler, C.; Hood, K.; Cooper, L.; Goossens, H. Clinical Microbiology and Infection 2009;15():S79 2009 DOI: 10.1111/j.1469-0691.2009.02857.x | Wrong publication type |
| Cabral, C., Ingram, J., Lucas, P. J., Redmond, N. M., Kai, J., Hay, A. D., & Horwood, J. (2016). Influence of Clinical Communication on Parents' Antibiotic Expectations for Children With Respiratory Tract Infections. *Annals of family medicine*, *14*(2), 141–147. https://doi-org.qe2a-proxy.mun.ca/10.1370/afm.1892 | Multiple reasons |
| Chalkidou, A., Lambert, M., Cordoba, G., Taxis, K., Hansen, M. P., & Bjerrum, L. (2023). Misconceptions and Knowledge Gaps on Antibiotic Use and Resistance in Four Healthcare Settings and Five European Countries-A Modified Delphi Study. *Antibiotics (Basel, Switzerland)*, *12*(9), 1435. https://doi-org.qe2a-proxy.mun.ca/10.3390/antibiotics12091435 | Multiple reasons |
| Chandy SJ, Mathai E, Thomas K, et al. Antibiotic use and resistance: perceptions and ethical challenges among doctors, pharmacists and the public in Vellore, SouthIndia. Ind J Med Ethic 2012;10(1):20-7 | Wrong population |
| Coenen, S., Van Royen, P., Vermeire, E., Hermann, I., & Denekens, J. (2000). Antibiotics for coughing in general practice: a qualitative decision analysis. *Family practice*, *17*(5), 380–385. https://doi-org.qe2a-proxy.mun.ca/10.1093/fampra/17.5.380 | Wrong population |
| Colliers, A., Coenen, S., Remmen, R., Philips, H., & Anthierens, S. (2018). How do general practitioners and pharmacists experience antibiotic use in out-of-hours primary care? An exploratory qualitative interview study to inform a participatory action research project. *BMJ open*, *8*(9), e023154. https://doi-org.qe2a-proxy.mun.ca/10.1136/bmjopen-2018-023154 | Multiple reasons |
| Elwyn, G., Gwyn, R., Edwards, A., & Grol, R. (1999). Is 'shared decision-making' feasible in consultations for upper respiratory tract infections? Assessing the influence of antibiotic expectations using discourse analysis. *Health expectations : an international journal of public participation in health care and health policy*, *2*(2), 105–117. https://doi-org.qe2a-proxy.mun.ca/10.1046/j.1369-6513.1999.00045.x | Multiple reasons |
| Gröndal H, Hedin K, Strandberg EL, André M, Brorsson A. Near-patient tests and the clinical gaze in decision-making of Swedish GPs not following current guidelines for sore throat - a qualitative interview study. BMC Fam Pract. 2015 Jul 4;16:81. doi: 10.1186/s12875-015-0285-y. PMID: 26141740; PMCID: PMC4491276. | Wrong phenomenon of interest |
| Gulliford, M. C., Charlton, J., Boiko, O., Winter, J. R., Rezel-Potts, E., Sun, X., Burgess, C., McDermott, L., Bunce, C., Shearer, J., Curcin, V., Fox, R., Hay, A. D., Little, P., Moore, M. V., & Ashworth, M. (2021). *Safety of reducing antibiotic prescribing in primary care: a mixed-methods study*. NIHR Journals Library. https://doi-org.qe2a-proxy.mun.ca/10.3310/hsdr09090 | Wrong population |
| Hayward, G. N., Moore, A., Mckelvie, S., Lasserson, D. S., & Croxson, C. (2019). Antibiotic prescribing for the older adult: beliefs and practices in primary care. *The Journal of antimicrobial chemotherapy*, *74*(3), 791–797. https://doi-org.qe2a-proxy.mun.ca/10.1093/jac/dky504 | Multiple reasons |
| Hart, A. M., Pepper, G. A., & Gonzales, R. (2006). Balancing acts: deciding for or against antibiotics in acute respiratory infections. *The Journal of family practice*, *55*(4), 320–325. | Wrong population |
| Horwood, J., Cabral, C., Hay, A. D., & Ingram, J. (2016). Primary care clinician antibiotic prescribing decisions in consultations for children with RTIs: a qualitative interview study. *The British journal of general practice : the journal of the Royal College of General Practitioners*, *66*(644), e207–e213. https://doi-org.qe2a-proxy.mun.ca/10.3399/bjgp16X683821 | Wrong population |
| Hosoglu, S., Classen, A. Y., & Akturk, Z. (2021). Antibiotic prescription in primary care from the perspective of family physicians: a qualitative study. *Journal of infection in developing countries*, *15*(8), 1117–1123. https://doi-org.qe2a-proxy.mun.ca/10.3855/jidc.13924 | Multiple reasons |
| Hounkpatin, H. O., Woods, C., Lown, M., Stuart, B., & Leydon, G. M. (2021). Understanding GPs' views and experiences of using clinical prediction rules in the management of respiratory infections: a qualitative study. *BJGP open*, *5*(4), BJGPO.2021.0096. https://doi-org.qe2a-proxy.mun.ca/10.3399/BJGPO.2021.0096 | Wrong phenomenon of interest |
| Hruza H, Velasquez T, Madaras-Kelly K, Fleming-Dutra K, Samore M, Butler J. 1888. Clinicians’ Beliefs, Knowledge, Attitudes, and Planned Behaviors on Antibiotic Prescribing in Acute Respiratory Infections. Open Forum Infect Dis. 2018 Nov 26;5(Suppl 1):S540–1. doi: 10.1093/ofid/ofy210.1544. PMCID: PMC6254035. | Wrong population |
| Krishnakumar, J., & Tsopra, R. (2019). What rationale do GPs use to choose a particular antibiotic for a specific clinical situation?. *BMC family practice*, *20*(1), 178. https://doi-org.qe2a-proxy.mun.ca/10.1186/s12875-019-1068-7 | Wrong phenomenon of interest |
| Manderson, L. Prescribing, care and resistance: antibiotic use in urban South Africa. *Humanit Soc Sci Commun* **7**, 77 (2020). https://doi-org.qe2a-proxy.mun.ca/10.1057/s41599-020-00564-1 | Wrong population |
| Mas-Dalmau, G., Pequeño-Saco, S., de la Poza-Abad, M., Borrell-Thió, E., Besa-Castellà, M., Alsina-Casalduero, M., Cuixart-Costa, L., Liroz-Navarro, M., Calderón-Gómez, C., Martí, J., Cruz-Gómez, I., & Alonso-Coello, P. (2023). Perceptions and attitudes regarding delayed antibiotic prescription for respiratory tract infections: a qualitative study. BMC primary care, 24(1), 204. https://doi-org.qe2a-proxy.mun.ca/10.1186/s12875-023-02123-4 | Wrong population |
| Moragas A, Molero JM, Bjerrum L, Llor C. General practitioners' opinions and perceptions about antibiotic use for respiratory tract infections in primary care. Aten Primaria. 2019 Aug-Sep;51(7):460-461. doi: 10.1016/j.aprim.2019.01.011. Epub 2019 Mar 26. PMID: 30922676; PMCID: PMC6837051. | Multiple reasons |
| Murphy, M.; Bradley, C. B.; Byrne, S. The culture of antibiotic prescribing in general practice in ireland-a qualitative study. Journal of Pharmacy and Pharmaceutical Sciences 2011;14(3):206s-207s | Multiple reasons |
| Mustafa, M., Wood, F., Butler, C. C., & Elwyn, G. (2014). Managing expectations of antibiotics for upper respiratory tract infections: a qualitative study. *Annals of family medicine*, *12*(1), 29–36. https://doi-org.qe2a-proxy.mun.ca/10.1370/afm.1583 | Wrong phenomenon of interest |
| Orero, A., Navarro, A., López, S., Olmo, V., Gonzalez, J., Prieto, J., & Grupo MUSA (2007). Conocimiento y actitud de los médicos de atención primaria en el tratamiento de las infecciones comunitarias [Knowledge and attitude of primary health care doctors in the treatment of community-acquired infections]. *Revista espanola de quimioterapia : publicacion oficial de la Sociedad Espanola de Quimioterapia*, *20*(3), 323–329. | Wrong study design |
| Saleh, H. A., Borg, M. A., Stålsby Lundborg, C., & Saliba-Gustafsson, E. A. (2022). General Practitioners', Pharmacists' and Parents' Views on Antibiotic Use and Resistance in Malta: An Exploratory Qualitative Study. *Antibiotics (Basel, Switzerland)*, *11*(5), 661. https://doi-org.qe2a-proxy.mun.ca/10.3390/antibiotics11050661 | Wrong population |
| Østergaard, M. S., Kjærgaard, J., Kristensen, M. M., Reventlow, S., Poulsen, A., Isaeva, E., Akylbekov, A., & Sooronbaev, T. (2018). Recurrent lower respiratory illnesses among young children in rural Kyrgyzstan: overuse of antibiotics and possible under-diagnosis of asthma. A qualitative FRESH AIR study. *NPJ primary care respiratory medicine*, *28*(1), 13. https://doi-org.qe2a-proxy.mun.ca/10.1038/s41533-018-0081-y | Multiple reasons |
| Patel, A.; Chaitoff, A.; Rothberg, M. B.; Hu, B.; Manne, M.; Pfoh, E.; Misra-Hebert, A. D. Understanding physician treatment decisions for the management of upper respiratory tract infections. Journal of General Internal Medicine 2017;32(2):S366 | Wrong publication type |
| Petursson P. (2005). GPs' reasons for "non-pharmacological" prescribing of antibiotics. A phenomenological study. *Scandinavian journal of primary health care*, *23*(2), 120–125. https://doi-org.qe2a-proxy.mun.ca/10.1080/02813430510018491 | Wrong population |
| Pradier, C.; Cavailler, P.; Rotily, M.; Keita-Perse, O.; Dunais, B.; Obadia, Y.; Dellamonica, P. General practitioner management of viral pharyngitis in children under 3 years of age. Medecine et Maladies Infectieuses 1999;29(3):154-159  1999 DOI: [10.1016/S0399-077X(99)80034-1](https://dx.doi.org/10.1016/S0399-077X(99)80034-1) | Wrong study design |
| Saliba-Gustafsson, E. A., Nyberg, A., Borg, M. A., Rosales-Klintz, S., & Stålsby Lundborg, C. (2021). Barriers and facilitators to prudent antibiotic prescribing for acute respiratory tract infections: A qualitative study with general practitioners in Malta. *PloS one*, *16*(2), e0246782. https://doi-org.qe2a-proxy.mun.ca/10.1371/journal.pone.0246782 | Wrong population |
| Sanchez GV, Roberts RM, Albert AP, Johnson DD, Hicks LA. Effects of knowledge, attitudes, and practices of primary care providers on antibiotic selection, United States. Emerg Infect Dis. 2014 Dec;20(12):2041-7. doi: 10.3201/eid2012.140331. PMID: 25418868; PMCID: PMC4257826. | Multiple reasons |
| Sharaf N, Al-Jayyousi GF, Radwan E, Shams Eldin SME, Hamdani D, Al-Katheeri H, Elawad K, Habib Sair A. Barriers of Appropriate Antibiotic Prescription at PHCC in Qatar: Perspective of Physicians and Pharmacists. Antibiotics (Basel). 2021 Mar 19;10(3):317. doi: 10.3390/antibiotics10030317. PMID: 33808517; PMCID: PMC8003259. | Wrong population |
| Spicer JO, Dukes AW, O’Neill KE, Herrera R, Hicks LA. 1890. Healthcare Professionals’ Knowledge, Attitudes, and Beliefs Regarding Factors That Contribute to Inappropriate Antibiotic Use. Open Forum Infect Dis. 2018 Nov 26;5(Suppl 1):S541. doi: 10.1093/ofid/ofy210.1546. PMCID: PMC6253794. | Wrong publication type |
| Strandberg, E. L., Brorsson, A., André, M., Gröndal, H., Mölstad, S., & Hedin, K. (2016). Interacting factors associated with Low antibiotic prescribing for respiratory tract infections in primary health care - a mixed methods study in Sweden. BMC family practice, 17, 78. https://doi-org.qe2a-proxy.mun.ca/10.1186/s12875-016-0494-z | Wrong population |
| van der Zande, M. M., Dembinsky, M., Aresi, G., & van Staa, T. P. (2019). General practitioners' accounts of negotiating antibiotic prescribing decisions with patients: a qualitative study on what influences antibiotic prescribing in low, medium and high prescribing practices. *BMC family practice*, *20*(1), 172. https://doi-org.qe2a-proxy.mun.ca/10.1186/s12875-019-1065-x | Wrong population |
| Vazquez-Lago, J. M., Lopez-Vazquez, P., López-Durán, A., Taracido-Trunk, M., & Figueiras, A. (2012). Attitudes of primary care physicians to the prescribing of antibiotics and antimicrobial resistance: a qualitative study from Spain. *Family practice*, *29*(3), 352–360. https://doi-org.qe2a-proxy.mun.ca/10.1093/fampra/cmr084 | Wrong population |
| Vennik, J., Eyles, C., Thomas, M., Hopkins, C., Little, P., Blackshaw, H., Schilder, A., Boardman, J., & Philpott, C. M. (2018). Management strategies for chronic rhinosinusitis: a qualitative study of GP and ENT specialist views of current practice in the UK. *BMJ open*, *8*(12), e022643. https://doi-org.qe2a-proxy.mun.ca/10.1136/bmjopen-2018-022643 | Wrong population |
| Williams, S. J., Halls, A. V., Tonkin-Crine, S., Moore, M. V., Latter, S. E., Little, P., Eyles, C., Postle, K., & Leydon, G. M. (2018). General practitioner and nurse prescriber experiences of prescribing antibiotics for respiratory tract infections in UK primary care out-of-hours services (the UNITE study). *The Journal of antimicrobial chemotherapy*, *73*(3), 795–803. https://doi-org.qe2a-proxy.mun.ca/10.1093/jac/dkx429 | Wrong phenomenon of interest |
| Wood, F., Phillips, C., Brookes-Howell, L., Hood, K., Verheij, T., Coenen, S., Little, P., Melbye, H., Godycki-Cwirko, M., Jakobsen, K., Worby, P., Goossens, H., & Butler, C. C. (2013). Primary care clinicians' perceptions of antibiotic resistance: a multi-country qualitative interview study. *The Journal of antimicrobial chemotherapy*, *68*(1), 237–243. https://doi-org.qe2a-proxy.mun.ca/10.1093/jac/dks338 | Wrong phenomenon of interest |
| Xia, R.; Willcox, M.; Moore, M.; Liu, J.; Hu, X. Y.; Fei, Y. Chinese doctors’ perspectives in managing antibiotic prescribing: a qualitative study. European Journal of Integrative Medicine 2021;48. DOI: 10.1016/j.eujim.2021.101893 | Multiple reasons |
| Yin, J., Dyar, O. J., Yang, P., Yang, D., Marrone, G., Sun, M., Sun, C., Sun, Q., & Lundborg, C. S. (2019). Pattern of antibiotic prescribing and factors associated with it in eight village clinics in rural Shandong Province, China: a descriptive study. *Transactions of the Royal Society of Tropical Medicine and Hygiene*, *113*(11), 714–721. https://doi-org.qe2a-proxy.mun.ca/10.1093/trstmh/trz058 | Multiple reasons |
| Zhang, Z., Zhan, X., Zhou, H., Sun, F., Zhang, H., Zwarenstein, M., Liu, Q., Li, Y., & Yan, W. (2016). Antibiotic prescribing of village doctors for children under 15 years with upper respiratory tract infections in rural China: A qualitative study. *Medicine*, *95*(23), e3803. https://doi-org.qe2a-proxy.mun.ca/10.1097/MD.0000000000003803 | Wrong population |
