# Supplementary material for: Barriers and enablers to using evidence-based antibiotic prescription guidelines in primary care: a qualitative systematic review and synthesis using the theoretical domains framework
Source: Implement Sci Commun. 2026 Feb 16;7:52. doi: 10.1186/s43058-025-00806-w (PMC13032215; doi:10.1186/s43058-025-00806-w)
Supplement: Supplementary file 4 — Supplementary Material 4. [file 43058_2025_806_MOESM4_ESM.docx]

Additional File 4 - Assessment of reporting criteria according to the guidance from CASP and COREQ

| Reporting Criteria | | | | | | Borek et al. 2022 | | Butler et al. 1998 | | Dallas et al. 2014 | Damoiseaux et al. 1999 | De Bock et al. 1994 | Dempsey et al. 2014 | Fletcher-Lartey et al. 2016 | Hedin et al. 2014 | Jaruseviciene et al. 2013 | Kadirhaz et al., 2024 | Kaae et al. 2017 | Kotwani et al. 2017 | Kumar et al. 2003 | Moe et al., 2021 | O’Doherty et al. 2019 | Patel et al. 2020 | Rutkovska et al., 2022 | Schubert et al., 2023 | Shen et al., 2023 | Simeoni et al. 2022 | Thaulow et al., 2023 | Tystrup et al. 2020 | Varonen et al. 2004 |
| --- | --- | --- | --- | --- | --- | --- | --- | --- | --- | --- | --- | --- | --- | --- | --- | --- | --- | --- | --- | --- | --- | --- | --- | --- | --- | --- | --- | --- | --- | --- |
| Aim | | Aim explicitly stated and relevant | | | | ✓ | | ✓ | | ✓ | ✓ | ✓ | ✓ | ✓ | ✓ | ✓ | ✓ | ✓ | ✓ | ✓ | ✓ | ✓ | ✓ | ✓ | ✓ | ✓ | ✓ | ✓ | ✓ | ✓ |
| Qualitative Approach | | Correct method used | | | | ✓ | | ✓ | | ✓ | ✓ | ✓ | ✓ | ✓ | ✓ | ✓ | ✓ | ✓ | ✓ | ✓ | ✓ | ✓ | ✓ | ✓ | ✓ | ✓ | ✓ | ✓ | ✓ | ✓ |
|  |  | Theoretical framework reported | | | | ✓ | | ✓ | | ✓ | 🗴 | 🗴 | ✓ | ✓ | ✓ | ✓ | ✓ | ✓ | ✓ | ✓ | ✓ | ✓ | ✓ | ✓ | ✓ | ✓ | ✓ | ✓ | ✓ | 🗴 |
| Design | | Design aligned to aims | | | | ✓ | | ✓ | | ✓ | ✓ | ✓ | ✓ | ✓ | ✓ | ✓ | ✓ | ✓ | ✓ | ✓ | ✓ | ✓ | ✓ | ✓ | ✓ | ✓ | ✓ | ✓ | ✓ | ✓ |
|  |  | Design appropriately justified | | | | ✓ | | ✓ | | ✓ | ✓ | ✓ | ✓ | ✓ | ✓ | ✓ | ✓ | ✓ | ✓ | ✓ | ✓ | ✓ | ✓ | ✓ | ✓ | ✓ | ✓ | ✓ | ✓ | ✓ |
| Recruitment | | A priori participant selection criteria | | | | ✓ | | ✓ | | ✓ | ✓ | ✓ | ✓ | ✓ | ✓ | ✓ | ✓ | ✓ | ✓ | ✓ | ✓ | ✓ | ✓ | ✓ | ✓ | ✓ | ✓ | ✓ | ✓ | ✓ |
|  |  | Recruitment strategy explained for replication | | | | ✓ | | ✓ | | ✓ | ✓ | 🗴 | ✓ | ✓ | 🗴 | ✓ | ✓ | ✓ | ✓ | ✓ | ✓ | 🗴 | ✓ | ✓ | ✓ | ✓ | ✓ | ✓ | 🗴 | ✓ |
|  |  | Purposive sampling used | | | | ✓ | | ✓ | | ✓ | ✓ | ✓ | ✓ | ✓ | ✓ | ✓ | ✓ | ✓ | ✓ | ✓ | ✓ | ✓ | ✓ | ✓ | ✓ | 🗴 | ✓ | ✓ | ✓ | ✓ |
| Data Collection | | Data collection explicit, enabling replication | | | | ✓ | | ✓ | | ✓ | 🗴 | ✓ | ✓ | ✓ | ✓ | ✓ | ✓ | ✓ | ✓ | ✓ | ✓ | ✓ | ✓ | ✓ | ✓ | ✓ | ✓ | ✓ | 🗴 | ✓ |
|  |  | Research setting identified | | | | 🗴 | | ✓ | | 🗴 | 🗴 | 🗴 | 🗴 | 🗴 | ✓ | 🗴 | ✓ | 🗴 | ✓ | ✓ | ✓ | ✓ | 🗴 | ✓ | ✓ | ✓ | 🗴 | ✓ | 🗴 | 🗴 |
|  |  | Focus group, interview, survey described | | | | ✓ | | ✓ | | ✓ | ✓ | ✓ | ✓ | ✓ | ✓ | ✓ | ✓ | ✓ | ✓ | ✓ | ✓ | ✓ | ✓ | ✓ | ✓ | ✓ | ✓ | ✓ | ✓ | ✓ |
|  |  | Data recorded and transcribed | | | | ✓ | | ✓ | | ✓ | 🗴 | ✓ | ✓ | ✓ | ✓ | ✓ | ✓ | 🗴 | ✓ | ✓ | ✓ | ✓ | ✓ | ✓ | ✓ | ✓ | ✓ | ✓ | ✓ | ✓ |
|  |  | Field notes taken | | | | 🗴 | | 🗴 | | 🗴 | 🗴 | 🗴 | 🗴 | 🗴 | ✓ | ✓ | 🗴 | 🗴 | 🗴 | 🗴 | 🗴 | 🗴 | 🗴 | 🗴 | ✓ | 🗴 | 🗴 | 🗴 | 🗴 | ✓ |
|  |  | Data saturation reported | | | | ✓ | | 🗴 | | ✓ | 🗴 | 🗴 | ✓ | ✓ | 🗴 | 🗴 | 🗴 | 🗴 | ✓ | ✓ | ✓ | ✓ | 🗴 | ✓ | ✓ | ✓ | ✓ | ✓ | 🗴 | ✓ |
| Researcher-participant relationship | | Interviewer level of influence of described | | | | 🗴 | | 🗴 | | 🗴 | 🗴 | 🗴 | 🗴 | 🗴 | 🗴 | 🗴 | 🗴 | 🗴 | 🗴 | 🗴 | 🗴 | 🗴 | 🗴 | 🗴 | ✓ | 🗴 | 🗴 | 🗴 | 🗴 | 🗴 |
|  |  | Interviewer identified and described | | | | ✓ | | 🗴 | | 🗴 | 🗴 | 🗴 | 🗴 | 🗴 | 🗴 | 🗴 | 🗴 | 🗴 | 🗴 | 🗴 | ✓ | ✓ | 🗴 | 🗴 | 🗴 | ✓ | ✓ | ✓ | 🗴 | ✓ |
| Ethics | | Ethics committee approval obtained | | | | ✓ | | ✓ | | 🗴 | 🗴 | ✓ | ✓ | ✓ | ✓ | ✓ | ✓ | ✓ | ✓ | 🗴 | ✓ | ✓ | ✓ | ✓ | ✓ | ✓ | ✓ | ✓ | ✓ | ✓ |
|  |  | Explanation of the study given | | | | ✓ | | 🗴 | | 🗴 | 🗴 | 🗴 | 🗴 | ✓ | ✓ | ✓ | ✓ | 🗴 | 🗴 | 🗴 | 🗴 | ✓ | 🗴 | 🗴 | ✓ | 🗴 | ✓ | 🗴 | 🗴 | 🗴 |
|  |  | Informed consent obtained | | | | ✓ | | 🗴 | | ✓ | 🗴 | 🗴 | ✓ | ✓ | ✓ | ✓ | ✓ | ✓ | ✓ | 🗴 | ✓ | ✓ | ✓ | ✓ | ✓ | ✓ | ✓ | ✓ | ✓ | 🗴 |
|  |  | Anonymous transcripts used | | | | ✓ | | 🗴 | | ✓ | 🗴 | 🗴 | 🗴 | 🗴 | 🗴 | 🗴 | 🗴 | 🗴 | 🗴 | 🗴 | ✓ | 🗴 | 🗴 | ✓ | ✓ | 🗴 | 🗴 | ✓ | 🗴 | 🗴 |
|  |  | Confidentiality described | | | | 🗴 | | 🗴 | | ✓ | 🗴 | 🗴 | 🗴 | 🗴 | ✓ | ✓ | 🗴 | ✓ | ✓ | 🗴 | 🗴 | 🗴 | 🗴 | ✓ | 🗴 | ✓ | 🗴 | ✓ | ✓ | 🗴 |
| Analysis | | Type (thematic, content) | | | | ✓ | | ✓ | | ✓ | 🗴 | 🗴 | ✓ | ✓ | ✓ | ✓ | ✓ | ✓ | ✓ | ✓ | ✓ | ✓ | ✓ | ✓ | ✓ | ✓ | ✓ | ✓ | ✓ | ✓ |
|  |  | Explicit steps of analysis process | | | | ✓ | | ✓ | | ✓ | 🗴 | ✓ | ✓ | ✓ | ✓ | ✓ | ✓ | ✓ | ✓ | ✓ | ✓ | ✓ | ✓ | ✓ | ✓ | ✓ | ✓ | ✓ | ✓ | ✓ |
|  |  | 2 researchers performed analysis | | | | ✓ | | ✓ | | ✓ | 🗴 | 🗴 | 🗴 | ✓ | ✓ | ✓ | ✓ | ✓ | ✓ | 🗴 | ✓ | ✓ | 🗴 | ✓ | ✓ | ✓ | ✓ | ✓ | 🗴 | ✓ |
|  |  | Quotations to support findings | | | | ✓ | | ✓ | | ✓ | 🗴 | 🗴 | ✓ | ✓ | ✓ | ✓ | ✓ | 🗴 | ✓ | ✓ | ✓ | ✓ | ✓ | ✓ | ✓ | ✓ | ✓ | ✓ | ✓ | ✓ |
|  |  | Contrary observations | | | | ✓ | | ✓ | | ✓ | 🗴 | ✓ | ✓ | ✓ | ✓ | 🗴 | ✓ | ✓ | ✓ | ✓ | ✓ | ✓ | ✓ | ✓ | 🗴 | 🗴 | 🗴 | ✓ | ✓ | 🗴 |
| Findings | | Explicit statement of findings | | | | ✓ | | ✓ | | ✓ | ✓ | ✓ | ✓ | ✓ | ✓ | ✓ | ✓ | ✓ | ✓ | ✓ | ✓ | ✓ | ✓ | ✓ | ✓ | ✓ | ✓ | ✓ | ✓ | ✓ |
|  |  | Credibility | | | | ✓ | | ✓ | | ✓ | 🗴 | 🗴 | ✓ | ✓ | ✓ | ✓ | 🗴 | 🗴 | ✓ | 🗴 | ✓ | ✓ | 🗴 | ✓ | ✓ | ✓ | ✓ | ✓ | 🗴 | 🗴 |
|  |  | Discussion linked to aims/literature | | | | ✓ | | ✓ | | ✓ | ✓ | ✓ | ✓ | ✓ | ✓ | ✓ | ✓ | ✓ | ✓ | ✓ | ✓ | ✓ | ✓ | ✓ | ✓ | ✓ | ✓ | ✓ | ✓ | ✓ |
|  |  | Strengths and limitations | | | | ✓ | | ✓ | | ✓ | ✓ | ✓ | ✓ | ✓ | ✓ | ✓ | ✓ | ✓ | ✓ | 🗴 | ✓ | ✓ | ✓ | ✓ | ✓ | ✓ | ✓ | ✓ | ✓ | ✓ |
| Value | | Contributions to knowledge base | | | | ✓ | | ✓ | | ✓ | ✓ | ✓ | ✓ | ✓ | ✓ | ✓ | ✓ | ✓ | ✓ | ✓ | ✓ | ✓ | ✓ | ✓ | ✓ | ✓ | ✓ | ✓ | ✓ | ✓ |
|  |  | Transferability (generalizability) | | | | ✓ | | 🗴 | | ✓ | ✓ | ✓ | 🗴 | ✓ | 🗴 | ✓ | ✓ | ✓ | ✓ | 🗴 | ✓ | ✓ | ✓ | ✓ | ✓ | ✓ | ✓ | ✓ | ✓ | ✓ |
|  |  | Recommendations for practices | | | | ✓ | | ✓ | | ✓ | ✓ | 🗴 | ✓ | ✓ | ✓ | ✓ | ✓ | ✓ | ✓ | ✓ | ✓ | ✓ | ✓ | 🗴 | ✓ | 🗴 | ✓ | 🗴 | ✓ | 🗴 |
|  |  | Recommendations for research | | | | ✓ | | 🗴 | | ✓ | ✓ | ✓ | ✓ | 🗴 | ✓ | 🗴 | ✓ | 🗴 | 🗴 | ✓ | 🗴 | 🗴 | 🗴 | 🗴 | ✓ | ✓ | ✓ | ✓ | ✓ | ✓ |
|  |  | |  |  |  | |  | | ✓ = component was reported, 🗴= component was not reported | | | | | | | | | | | | | | | | | | | | | |
