# Supplementary material for: Barriers and enablers to using evidence-based antibiotic prescription guidelines in primary care: a qualitative systematic review and synthesis using the theoretical domains framework
Source: Implement Sci Commun. 2026 Feb 16;7:52. doi: 10.1186/s43058-025-00806-w (PMC13032215; doi:10.1186/s43058-025-00806-w)
Supplement: Supplementary file 5 — Supplementary Material 5. [file 43058_2025_806_MOESM5_ESM.docx]

Additional File 5 – Summary of Findings

Barriers and enablers to FP guideline-based antibiotic prescribing for URTIs in primary care

| TDF Domain | Barrier, Enabler, Neutral | Theme | Sample Quote | Contributing  studies #  Studies (sample),  references | Quality of evidence |
| --- | --- | --- | --- | --- | --- |
| Knowledge | Barrier | Lack of knowledge or misinformation on antibiotics (e.g., when to prescribe antibiotics and how they work) and antibiotic resistance may lead to unnecessary antibiotic prescribing for URTIs | “Further probing revealed that many GDs and FPs assumed that newer combinations or variants of the same class were ‘newer antibiotics.’” (Author Interpretation - Kotwani, 2017)  “However, most felt that this evidence was not “watertight” and that antibiotics may help some patients.” (Author Interpretation - Butler, 1998) | 9(193)  ^39–41,44,48–52^ | High  No/Very minor concerns regarding methodological limitations, Minor concerns regarding coherence (ambiguous data present in <25% of the supporting data), No/Very minor concerns regarding adequacy, and Minor concerns regarding relevance (<25% of the supporting data has indirect relevance to the review (i.e., conditions outside the inclusion criteria). |
|  | Barrier | Despite being aware of URTI antibiotic prescribing guidelines, some FPs continue to prescribe, incorrectly believe they are following them or believe they are not well-developed | “All FPs that we interviewed said they were aware of the guidelines but only the nine adherent FPs were able to recapture them correctly” (Author Interpretation - Hedin, 2014) | 3(67)  ^46,53,60^ | Moderate  No/Very minor concerns regarding methodological limitations, Moderate concerns regarding coherence (25%-50% of supporting data has ambiguous data), No/Very minor concerns regarding adequacy, and Minor concerns regarding relevance (<25% of the supporting data is of indirect relevance). |
|  | Barrier | Lack of knowledge about URTI antibiotic prescribing guidelines can lead to inappropriate antibiotic prescribing for URTIs | “However, most FPs were unable to recite either the guidelines or the Centor criteria correctly.” (Author Interpretation - Hedin, 2014) | 3(73)  ^39,46,60^ | Moderate  No/Very minor concerns regarding methodological limitations, No/Very minor concerns regarding coherence, No/Very minor concerns regarding adequacy, and Moderate concerns regarding relevance (25-50% of the data may be indirectly relevant to the research question). |
|  | Barrier | Lack of knowledge or misconceptions about infections can lead to more unnecessary antibiotic prescribing for URTIs | “Many physicians believe that many of the bronchitises are caused by bacterial infections especially when the sputum is green, which is not really true.” (Direct Quote - Dempsey, 2014) | 7(153)  ^44,46,56,57,59–61^ | Moderate  No/Very minor concerns regarding methodological limitations, Moderate concerns regarding coherence (50% of the data is unclear or vague), Minor concerns regarding adequacy (the data comes from multiple studies in different settings and varying sample sizes and <25% of the supporting data is too superficial), and No/Very minor concerns regarding relevance. |
|  | Barrier | Lack of knowledge of personal URTI antibiotic prescribing rates can lead to inappropriate antibiotic prescribing for URTIs | “I’d be more interested in my own prescribing in comparison to the other doctors within the practice […]” (Direct Quote - Borek, 2022) | 2(41)  ^39,49^ | Very low  Minor concerns regarding methodological limitations (data comes from two studies with moderate rigour), Moderate concerns regarding coherence (unclear if some of the data supports the review finding), Moderate concerns regarding adequacy(the data is not superficial but is from only two studies), and Serious concerns regarding relevance the supporting has some information of partial relevance (i.e., conditions outside of the inclusion criteria). |
|  | Enabler | Knowledge of antibiotics and antibiotic resistance can lead to less antibiotic prescribing for URTIs | “I think, as a FP over the last four or five years, I’ve become more conscious of changes in antibiotic prescribing in general practice as a whole. And I think that the conditions we used to prescribe more antibiotics for, such as sore throat, in the past we do less so now because of research which has become well publicized. I am more reluctant to prescribe, so I suppose I would say that I try to reserve antibiotics for when I feel I’m likely to be dealing with bacterial sore throat” (Direct Quote - Kumar, 2003)  “FPs interviewed were aware that antibiotics are being used inappropriately for non-bacterial minor illness such as ARTIs. FPs believed that this can lead to AMR. The FPs were also aware that over the last thirty years, there has been no new class of antibiotic introduced and that AMR is a real public health threat.” (Author Interpretation - O’Doherty, 2019) | 5(97)  ^39,40,48,51,53^ | High  No/Very minor concerns regarding methodological limitations, No/Very minor concerns regarding coherence, No/Very minor concerns regarding adequacy, and Minor concerns regarding relevance (some of the supporting data (< 25%) is of indirect relevance to the research question (i.e., one condition outside of inclusion criteria). |
|  | Enabler | Knowledge about URTI antibiotic prescribing guidelines helps FP prescribe appropriately | “The consistent theme that emerged regarding guidelines and antibiotic prescribing for acute bronchitis was that all participants agreed with guidelines stating that antibiotics are not indicated for acute bronchitis” (Author Interpretation - Dempsey, 2014) | 4(70)  ^41,44,52,60^ | Moderate  No/Very minor concerns regarding methodological limitations, No/Very minor concerns regarding coherence, No/Very minor concerns regarding adequacy, and Moderate concerns regarding relevance (some of the supporting data (25-50%) is of indirect relevance). |
|  | Enabler | Knowledge about infections can lead to less antibiotic prescribing for URTIs | “I mean there is plenty of evidence now to suggest that most of these cases are viral and self limiting and get better” (Direct Quote - Kumar, 2003) | 4(99)  ^43,49,51,60^ | Low  No/Very minor concerns regarding methodological limitations, Minor concerns regarding coherence, Serious concerns regarding adequacy (the data is based on a few studies with superficial data), and Moderate concerns regarding relevance (25-50% of the supporting data may be indirectly relevant to the research question (i.e. one condition outside of inclusion criteria). |
| Skills | Barrier | Lack of communication skills can lead to prescribing | "Also it can be difficult to elicit the patient’s views on the benefits of antibiotics and whether the main purpose of their visit is for medical assessment of their illness and symptomatic  treatment only if that is all that is indicated. "(Author Interpretation - O’Doherty, 2019) | 1(13)  ^53^ | Low  No/Very minor concerns regarding methodological limitations, Moderate concerns regarding coherence (lack of ability to assess contradictory data), Serious concerns regarding adequacy (the data is from a single study, with a small sample and the data is thin), and No/Very minor concerns regarding relevance. |
|  | Barrier | Lack of clinical skills can increase antibiotic prescribing for URTIs | “**Lack of skill** and …thus hampered adherence.” (Author Interpretation - Tystrup, 2020) | 1(29)  ^60^ | Very low  Moderate concerns regarding methodological limitations(the data comes from a single study with moderate rigour), Moderate concerns regarding coherence (the data comes from a single study and is not well explored), Serious concerns regarding adequacy(the data is superficial and is from one study with a small sample size), and Serious concerns regarding relevance (the data may be of indirect relevance since the study included a condition outside the inclusion criteria). |
|  | Enabler | Communication skills help reduce antibiotic prescribing for URTIs | “Learning communication skills and confidence to build therapeutic relationships was perceived to improve [prescribing] also…”(Author Interpretation - Dallas, 2014) | 1(17)  ^41^ | Low  No/Very minor concerns regarding methodological limitations, Serious concerns regarding coherence(the data comes from a single study and is not well explored), Serious concerns regarding adequacy (the data is from a single study, with a small sample, and the data is superficial), and No/Very minor concerns regarding relevance. |
| Social and Professional Role and Identity | Barrier | FPs think prioritizing patient care is their priority, not antibiotic resistance or guideline-based care which can lead to unnecessary antibiotic prescribing. | “So if I think I’m treating the whole patient and not just the virus then I feel better about giving the antibiotic here— because there is a holistic duty here.” (Direct Quote - Kumar 2003)  “For many FPs, the guidelines for acute tonsillitis did not seem to be compatible with existing values.” (Author Interpretation - Tystrup, 2020) | 7(168)  ^40,46,51,55,58–60^ | High  No/Very minor concerns regarding methodological limitations, Minor concerns regarding coherence(threats present (competing theories) in <25% of the supporting data), Minor concerns regarding adequacy(the data comes from multiple studies in different settings and varying sample sizes and <25% of the supporting data is too superficial), and Minor concerns regarding relevance (one of the supporting data (< 25%) is of partial relevance (one of the supporting data (< 25%) is of indirect relevance to the research question (one study includes a condition outside the scope)). |
|  | Barrier | FPs reported that supervisor priorities can lead them to overprescribe antibiotics for URTIs | “FP registrars have been sent emails to say that they should prescribe antibiotics, because that’s what patients expect, but that’s coming down from the older generations.” (Direct Quote - Dallas, 2014) | 1(17)  ^41^ | Low  No/Very minor concerns regarding methodological limitations, Moderate concerns regarding coherence( the data is not well explored in different contexts), Serious concerns regarding adequacy (data is thin and comes from a single study), and No/Very minor concerns regarding relevance |
|  | Barrier | FPs report prescribing more antibiotics for URTIs later in their career | "I’d say I prescribe antibiotics for three in every 10 I see. When I first started, I prescribed very little—it was very low, it was actually lower than it is today. I was very strict and just stuck to the facts. Now it [antibiotic prescribing] has increased. My antibiotic prescribing for sore throat has waxed and waned over the years, and at the moment it is less than it’s been at other times but not as low as when I started…” (Direct Quote - Kumar, 2003) | 2(61)  ^40,51^ | Very low  No/Very minor concerns regarding methodological limitations, Moderate concerns regarding coherence (some of the data is unclear and may not be congruent with the research finding), Moderate concerns regarding adequacy (the data is not thin but comes from one country), and Serious concerns regarding relevance ( the data may be of partial relevance to the research question as it only relates to one URTI). |
|  | Barrier | Locums can be unconnected to clinics or teams and may have less influence on antibiotic prescribing for URTIs | “… you’re not part of the team and they don’t make you feel part of a team, you’re just there to come in and cover the session and that’s all you’re there to do. You’re not involved in discussions about prescribing or the processes in the practice […] it’s not really my role as a locum to get involved in trying to change processes that don’t seem to be working.” (Direct Quote – Borek, 2022) | 1(19)  ^39^ | Very Low  Moderate concerns regarding methodological limitations (the data comes from a single study with moderate rigour), Moderate concerns regarding coherence (the data comes from a single study and may have plausible alternatives or competing theories), Moderate concerns regarding adequacy ( the data is rich but comes from a single study), and Serious concerns regarding relevance (the data comes from a single study and may be of indirect relevance to the research question as it includes conditions outside the inclusion criteria). |
|  | Enabler | FPs think it as their responsibility to not overprescribe antibiotics for URTIs | ‘It’s very much an individual FP responsibility really… all FPs should understand what appropriate antibiotic prescribing is, that’s real basic bread and butter general practice. (Direct Quote - Borek, 2022)  "I now follow what is good medicine as opposed to what just makes people happier. I’m not here to make the patient happy because they have come here; I’m here to advise on what’s the best thing for them…” (Direct Quote - Kumar, 2003) | 6(120)  ^39,41,44,51,52,58^ | High  No/Very minor concerns regarding methodological limitations, No/Very minor concerns regarding coherence, No/Very minor concerns regarding adequacy, and Minor concerns regarding relevance (some of the supporting data (< 25%) is of indirect relevance (one study included a condition not in the scope of this review)). |
|  | Enabler | Locums may be in a better position to positively influence prescribing practices of clinics | “However, participants also described how seeing unfamiliar patients may put locums in a better position to suggest a ‘new’ no-antibiotic approach, and that locums might be less concerned about potential negative impact of not prescribing on doctor–patient relationship” (Author Interpretation - Borek, 2022) | 1(19)  ^39^ | Very Low  Moderate concerns regarding methodological limitations (the data comes from a single study with moderate rigour), Moderate concerns regarding coherence (the data may have plausible alternatives or competing theories that are not congruent to the research finding), Moderate concerns regarding adequacy ( the data comes from a single study and may not be representative of all locum FPs), and Serious concerns regarding relevance (the data comes from a single study and may be of indirect relevance to the research question because it includes conditions outside of the inclusion criteria for this review). |
|  | Neutral/  Unclear | FP identified themselves as high or low prescribers | “Only one general practitioner said he prescribed for half of those consulting, and he labelled himself as a high prescriber.” (Author Interpretation - Kumar, 2003)  "I’ve had colleagues say to me, I tend to be stingier with antibiotics than other people are. I think that people prescribe more than I do." (Direct Quote - Patel, 2019) | 2(69)  ^51,54^ | N/A |
|  | Neutral/  Unclear | FPs reported other staff contributing to overprescription of antibiotics | “I don’t think FPs contribute in any significant way, not really, and I think we are being targeted unfairly. Most FPs try desperately hard not to prescribe antibiotics, and it’s really a fallacy to say we overprescribe. For instance, look at penicillin; look at how long this has been around. OK, tell me why it still works in the community if we’re supposed to be causing resistance through its overuse. My argument is that I saw much more co-amoxiclav being used in hospitals than I ever did in general practice. And now we hear about antibiotics being used willy-nilly in farming, so looking at our prescribing of penicillin for sore throat is nonsense to me” (Direct Quote - Kumar, 2003) | 4(119)  ^44,49–51^ | N/A |
|  | Neutral/  Unclear | FPs identified as tension between mitigating risks of antibiotic resistance and prioritizing patient care | “It appears that the private health service model presents a potential conflict for the FP who has a duty to provide healthcare based on best evidence but must also satisfy private patients’ expectations in order to retain them.” (Author Interpretation - O’Doherty, 2019)  “Many physicians felt tension between their perceived Professional Role to prioritize doing what was right for the patient while also mitigating the population-level risks of antimicrobial resistance.” (Author Interpretation - Simeoni, 2022) | 7(168)  ^41,42,45,46,53,55,58^ | N/A |
| Beliefs about capabilities | Barrier | Lack of confidence in their ability and/or knowledge to diagnose or manage URTIs correctly can led to unnecessary antibiotic prescribing | “I think in the back of my mind “I don’t know everything, maybe they’ve seen something like this before and it has been bacterial.” I guess I’m just assuming that they have some kind of clinical knowledge that I don’t.” (Direct Quote - Dempsey, 2014)  “In episodic settings or situations with no prior relationship and increased time pressure, **many physicians reported feeling less confident in their ability to avoid antibiotics**.” (Direct Quote - Simeoni, 2022) | 8(207)  ^41,45,50–52,54,56,58^ | High  No/Very minor concerns regarding methodological limitations, No/Very minor concerns regarding coherence, Minor concerns regarding adequacy (one study had superficial data) , and Minor concerns regarding relevance (some of the supporting data (< 25%) is indirectly relevant) |
|  | Enabler | Confidence in their abilities and knowledge helped FPs manage URTIs without prescribing antibiotics | “I mean, maybe, five years ago, I would have been like, okay, fine, and now I’m like, no, I’m not going to give you antibiotics if it doesn’t make sense. I feel more confident to be firm in my convictions and my assessment of patients.”(Direct Quote - Simeoni, 2022) | 6(104)  ^41,49,52,55,58,59^ | High  (No/Very minor concerns regarding methodological limitations, No/Very minor concerns regarding coherence, No/Very minor concerns regarding adequacy, and No/Very minor concerns regarding relevance) |
|  | Neutral/  Unclear | FP confidence can help reduce inappropriate antibiotic prescribing for URTIs | “Interviewees described antibiotic prescribing as complex clinical decisions influenced by individual level factors, such as the FP’s experience, skills, **confidence**, the patient’s clinical presentation (for example, symptoms), and expectations/behaviour.” (Author Interpretation - Borek, 2022) | 2(59)  ^39,51^ | N/A |
| Optimism | Barrier | Pessimistic views on being a doctor | “There are limited (diagnostic) facilities at primary care facilities, so it is not possible to directly determine whether URTIs are bacterial or viral…. It’s not like here (primary care facilities), where it’s all about fear and responsibility. **It’s really hard to be a doctor these days…**” (Direct Quote – Shen, 2023) | 1(30)  ^57^ | Low  No/Very minor concerns regarding methodological limitations, Serious concerns regarding coherence (the data comes from a single study and did not assess for contradictory cases or variation in contexts), Serious concerns regarding adequacy ( the data comes from a single study and is very thin), and No/Very minor concerns regarding relevance |
|  | Barrier | Pessimistic about guidelines for antibiotic URTI prescribing being implemented | “Guidelines would not be implemented in everyday practice, let us face it, that is not going to happen. Here we do not have guideline-guided medicine.” (Direct Quote – Rutkovska, 2022) | 1(8)  ^55^ | Very low  Moderate concerns regarding methodological limitations (the data comes from a single study with moderate rigour), Serious concerns regarding coherence (the data comes from a single study and did not assess for contextual variation or contradictory cases), Serious concerns regarding adequacy (the data comes from a single study with a low N and is thin), and Serious concerns regarding relevance (the data comes from a single study and focuses only on one URTI) |
| Beliefs about consequences | Barrier | Prescribing antibiotics for URTIs helps to avoid missing complications or something significant | “… Some people I have to give antibiotics, they’re very sick, their temperature’s up. So there, I’m not waiting for academic research.. Infection can kill; an antibiotic has never killed anybody. So I choose to prescribe antibiotic…” (Direct Quote - Fletcher-Lartey 2016)  “Prescribing antibiotics is [like] some kind of insurance ... You can say it’s like insurance against complications. It’s also protection from groundless discussions, inspectors, audits, etc. (Lithuania)” (Direct Quote - Jaruseviciene, 2013)  “When you give a prescription of antibiotics to a patient with probable sinusitis, you feel safe, you can sleep your nights in peace” (Direct Quote - Varonen, 2004) | 18(408)  ^39,41–45,47–54,56–58,61^ | High  Minor concerns regarding methodological limitations, No/Very minor concerns regarding coherence, Minor concerns regarding adequacy (data comes from multiple studies in different settings and varying sample sizes and <25% of the supporting data is too superficial), and Minor concerns regarding relevance (some of the supporting data (< 25%) is of partial relevance (i.e., contained a condition outside of our inclusion criteria)) |
|  | Barrier | It takes too long to explain why antibiotic are not prescribed for URTIs | “You spend 15 minutes trying to educate them, when they will go out disillusioned, come back the next day and see someone else, making you feel 5 minutes would be better spent just giving them a prescription and getting rid of them.” (Direct Quote - Butler, 1998) | 3(78)  ^40,41,51^ | High  (No/Very minor concerns regarding methodological limitations, No/Very minor concerns regarding coherence, Minor concerns regarding adequacy (the data for the finding comes <25% of the included studies and only two countries) , and No/Very minor concerns regarding relevance) |
|  | Barrier | Prescribing antibiotics for URTIs does not impact antibiotic resistance and can have benefits for patient care | “I guess I probably tend to think “oh look it’s just amoxicillin”. Like, amoxicillin is not useful for anything anyway. You know, you’re not going to be using that in intensive care to treat people with multiple resistant things. Is it really going to do you that much harm just to give this patient a bit of amoxicillin?” (Direct Quote - Dallas, 2014)  “I don’t think FPs contribute in any significant way, not really, and I think we are being targeted unfairly. Most FPs try desperately hard not to prescribe antibiotics, and it’s really a fallacy to say we overprescribe. **For instance, look at penicillin; look at how long this has been around. OK, tell me why it still works in the community if we’re supposed to be causing resistance through its overuse.** My argument is that I saw much more co-amoxiclav being used in hospitals than I ever did in general practice. And now we hear about antibiotics being used willy-nilly in farming, so looking at our prescribing of penicillin for sore throat is nonsense to me” (Direct Quote - Kumar, 2003) | 9(195)  ^40,41,43–45,50,51,55,56^ | High  No/Very minor concerns regarding methodological limitations, No/Very minor concerns regarding coherence, No/Very minor concerns regarding adequacy, and Minor concerns regarding relevance (some of the supporting data (< 25%) is of partial relevance (i.e., contained a condition outside of our inclusion criteria)) |
|  | Barrier | Prescribe antibiotics for URTIs to protect their business | ‘Well most of the time I will have to survive with my practice, so patient’s expectation is a big thing for me. I cannot make them angry and drive them away, [laughing] I mean I will be out of my job next day, so I will have to follow the trend” (Direct Quote - Fletcher-Lartey, 2016) | 5(148)  ^40,45,50,57,58^ | High  No/Very minor concerns regarding methodological limitations, No/Very minor concerns regarding coherence, No/Very minor concerns regarding adequacy, and Minor concerns regarding relevance (one study includes a condition outside of our inclusion criteria) |
|  | Barrier | Prescribe to not risk or preserve their doctor-patient relationships | “Many were concerned to preserve and build relationships with their patients, and it was not worth jeopardizing this “for the sake of a prescription for penicillin V.” (Direct Quote - Butler, 1998) | 6(106)  ^40,45,53,55,56,58^ | High  No/Very minor concerns regarding methodological limitations, No/Very minor concerns regarding coherence, No/Very minor concerns regarding adequacy, and No/Very minor concerns regarding relevance |
|  | Barrier | Fear that patients will find out about falsification of medical records can lead to unnecessary antibiotic prescribing for URTIs | “Sometimes the patient can take his medical records, read them and say, ‘I haven’t taken this medication – it was not prescribed to me.’ (Russia)” (Direct Quote - Jaruseviciene, 2013) | 1(51)  ^47^ | Very Low  Moderate concerns regarding methodological limitations, Moderate concerns regarding coherence (the data fits the review finding, but the study did not assess for disconfirming cases), Moderate concerns regarding adequacy (the data comes from a single study that includes only two countries), and Serious concerns regarding relevance (the data comes from a single study including two countries, and the finding is likely country-specific which is not as relevant to our research question) |
|  | Barrier | Following guidelines for antibiotic prescribing for URTIs leads to more quinsy | “They adhered to the policy for almost a year, during which they observed an unprecedented rise in the number of patients with quinsy (diagnosis confirmed on admission to hospital). They linked this rise to their policy and returned to prescribing antibiotics for the severest sore throat symptoms and subsequently saw cases of quinsy fall.” (Author Interpretation - Jaruseviciene, 2013) | 1(40)  ^51^ | Very Low  Moderate concerns regarding methodological limitations (the data comes from a single study of moderate rigour), Serious concerns regarding coherence ( the data comes from a single study and did not assess for contradictory cases or contextual variation), Moderate concerns regarding adequacy (the data comes from a single study on a broad phenomenon, in a single setting but the sample size is large), and Serious concerns regarding relevance (the data is for only pharyngitis and may not be applicable to other URTIs) |
|  | Barrier | Using delayed prescribing can result in negative impacts on the doctor-patient relationship and still leads to unnecessary antibiotic prescribing for URTIs | “However, another general practitioner thought leaving the prescription in reception for the patient to collect after 48 hours was paternalistic, diminished the trust between patient and doctor, and was ultimately disempowering to the patient.” (Author Interpretation - Kumar, 2003) | 1(40)  ^51^ | Very Low  Moderate concerns regarding methodological limitations (the data comes from a single study with moderate rigour), Moderate concerns regarding coherence (unclear whether they could assess the presence of contradictory data), Moderate concerns regarding adequacy (he data comes from a single study on a broad phenomenon, in a single setting but the sample size is large), and Serious concerns regarding relevance (the data comes from a single study and focused on one URTI) |
|  | Enabler | Following guidelines protects FPs from medicolegal issues and grounds management decision in evidence based medicine. | “If you treat a patient as per the guidelines, even if the treatment fails we have a definite ground to stand on and I think that is more safe rather than choosing something else outside the guidelines.” (Direct Quote - Dallas, 2014) | 3(76)  ^41,47,55^ | High  No/Very minor concerns regarding methodological limitations, No/Very minor concerns regarding coherence, Minor concerns regarding adequacy (the data comes from three studies but includes four countries), and No/Very minor concerns regarding relevance |
|  | Enabler | Overprescribing antibiotics has numerous negative consequences for URTI care and antibiotic resistance | “While almost all acknowledged that prescribing might reduce workload in the short term (a quick consultation and reduction of the risk of an after hours visit), the vast majority recognized that prescribing antibiotics probably increased workload in the long term.” (Author Interpretation - Butler, 1998)  “Many registrars acknowledged the potential adverse consequences of overprescription of antibiotics.” (Author Interpretation - Dallas, 2014) | 6(94)  ^40,41,43,45,48,52^ | High  No/Very minor concerns regarding methodological limitations, No/Very minor concerns regarding coherence, Minor concerns regarding adequacy (some of the supporting data is superficial but is from multiple studies, and a several different countries that vary in socioeconomic factors), and No/Very minor concerns regarding relevance. |
|  | Enabler | Not prescribing antibiotics for URTIs does not negatively impact or can benefit the doctor-patient relationship | “Withholding antibiotics is not the worst thing I do in terms of the doctor-patient relationship. After all, my patients know me well enough by now. They know I base my advice by considering their story alongside the medical evidence. The sore throat doesn’t exist in isolation—often I’ve seen the same person with blood pressure, depression, diabetes, the list goes on—so, no, my relationship with my patients isn’t that fragile.” (Direct Quote - Kumar, 2003) | 3(76)  ^39,41,51^ | Moderate  No/Very minor concerns regarding methodological limitations, Moderate concerns regarding coherence (threats present in 25-50% of the supporting data), Minor concerns regarding adequacy (is predominately from a single country), and Minor concerns regarding relevance (some of the supporting data (< 25%) is of partial relevance). |
|  | Enabler | Using delayed prescription reduces consultation time and unnecessary antibiotic prescriptions for URTIs | “...delayed prescribing was regarded positively, and general practitioners thought it could be used to manage diagnostic uncertainty, to reassure the patient, to prevent reattendance, to reduce the likelihood of a patient taking the antibiotic, and to shorten consultation time.” (Author Interpretation - Kumar, 2003) | 1(40)  ^51^ | Very Low  Moderate concerns regarding methodological limitations (the data comes from a single study with moderate rigour), Serious concerns regarding coherence( the data comes from a single study and did not assess the phenomenon of interest in different contexts), Moderate concerns regarding adequacy (the data comes from a single study and a single setting), and Serious concerns regarding relevance (the data comes from a single study that focuses on one URTI). |
|  | Enabler | It doesn’t take long to explain why antibiotics are not necessary for URTIs | "No. It doesn’t take long to give them my here’s why-you-don’t-need and that takes 30 seconds to a minute even twice." (Direct Quote - Patel, 2019) | 1(29)  ^54^ | Very Low  Moderate concerns regarding methodological limitations (the data comes from a single study with moderate rigour), Serious concerns regarding coherence ( the data comes from a single study; therefore, it is unlikely that it is fully representative of all clinical practice), Serious concerns regarding adequacy(the data comes from a single study and the study did not assess the phenomenon of interest in different contexts), and No/Very minor concerns regarding relevance. |
|  | Neutral/Unclear | Antibiotic prescribing for URTIs can have significant impact on the therapeutic power of the doctor-patient relationship | “Almost every practitioner mentioned that prescribing decisions for these conditions could have an important impact on the therapeutic power of the doctor-patient relationship” (Author Interpretation - Butler, 1998) | 1(21)  ^40^ | N/A |
| Reinforcement | Barrier | Previous negative experiences influence FPs to prescribe antibiotics for URTIs | “The general practitioner who said he prescribed antibiotics for half of his patients with sore throat explained his “high” prescribing was grounded in an experience when he withheld antibiotics and the patient subsequently developed streptococcal septicaemia.” (Author Interpretation - Kumar, 2003) | 4(104)  ^41,42,46,51^ | Moderate  Moderate concerns regarding methodological limitations (25-50% of the supporting data comes from studies with low methodological rigour), No/Very minor concerns regarding coherence, Minor concerns regarding adequacy (the data comes from multiple studies in different settings and varying sample sizes and <25% of the supporting data is too superficial), and No/Very minor concerns regarding relevance |
|  | Barrier | Experience prescribing antibiotics for URTIs reinforced their beliefs that antibiotics are an effective treatment for URTIs | **“**However, I feel I am prescribing in response to what people are actually like, and you know not all will be satisfied, or you know some people will not be satisfied unless they get their antibiotic and I know who those people are, so when they come in I give them antibiotics. **I think research into this has been helpful, but I’ve learnt a lot from the hundreds of patients I’ve seen with sore throats too. People aren’t always as research would have them"** (Direct Quote - Kumar, 2003) | 2(70)  ^51,57^ | Very Low  No/Very minor concerns regarding methodological limitations, Moderate concerns regarding coherence (50% of the supporting data is vaguely described and therefore may not clearly support this review finding), Moderate concerns regarding adequacy (50% of the data lacks richness to fully interpret the meaning and the data only comes from two studies), and Moderate concerns regarding relevance (50% of the data is of partial relevance to the review's population of interest). |
|  | Barrier | Hard to change habit of prescribing antibiotics for URTIs | “...doctors that have been practicing for a long time, its sort of what they’ve always done…” (Direct Quote - Dempsey, 2014) | 3(63)  ^42,44,60^ | Very low  Moderate concerns regarding methodological limitations (because 25-50% of the supporting data comes from studies with low methodological rigour), No/Very minor concerns regarding coherence, Serious concerns regarding adequacy( the data comes from only a few studies and >50% of the supporting data is too superficial), and Moderate concerns regarding relevance(between 25% to 50% is of partial relevance) |
|  | Enabler | Experience has allowed FPs to become better at reducing antibiotic prescribing for URTIs | "doctors learn over a period of time about what to prescribe in what cases and they gather expertise with time” (Direct Quote - Kotwani, 2017) | 4(94)  ^41,50,52,58^ | Moderate  No/Very minor concerns regarding methodological limitations, No/Very minor concerns regarding coherence, Minor concerns regarding adequacy (the data comes from multiple studies in different settings and varying sample sizes and <25% of the supporting data is too superficial), and Moderate concerns regarding relevance (some of the supporting data (25-50%) is of partial relevance) |
|  | Enabler | Experiences with antibiotic resistance have influenced FPs to reduce antibiotic prescribing | “I did do an elective overseas … so I have seen a culture that has been able to buy amoxicillin over the counter and developed complete resistance to it, to the point where nobody found any benefit from it anymore over there … that does shape the way I prescribe for basic upper respiratory tract infections and things, that I won’t just hand it out for every patient that comes along because I’ve seen the effects of it being so widely used.’” (Direct Quote - Dallas, 2014) | 1(17)  ^41^ | Low  Moderate concerns regarding methodological limitations (the data is from a single study with moderate rigour), No/Very minor concerns regarding coherence, Moderate concerns regarding adequacy(it is a single study, with a fairly large sample, and the data is mostly not superficial), and No/Very minor concerns regarding relevance) |
|  | Neutral/Unclear | Experience informs FP antibiotic prescribing for URTIs | “Prescribing based on self-experience was frequently followed.” (Author Interpretation - Kotwani, 2017) | 5(129)  ^39,41,50,51,55^ | N/A |
| Intentions | Barrier | Not willing to change or doing things the way they want to | “I think when I was a young fiery FP trainee I used to try and not give antibiotics and now I’m softening and I give antibiotics more than I used to . . . **I’m quite well aware of the lack of firm evidence that antibiotics treat URTIs and that in terms of evidence based medicine we overprescribe antibiotics, but my own view is that I don’t really care .** . .” (Direct Quote - Butler, 1998) | 1(21)  ^40^ | Very Low  Moderate concerns regarding methodological limitations (the data is from a single study with moderate rigour), Moderate concerns regarding coherence (the data comes from a single study and did not assess for the variation in different contexts), Serious concerns regarding adequacy (the data comes from a single study with a small sample size and the data is thin), and Moderate concerns regarding relevance (the data comes from a single study that focuses on one URTI) |
|  | Enabler | Try to do things the “right way” and follow guidelines | “Most attempted to explain to patients that viruses are not responsive to antibiotics, both to justify not prescribing and to express their professional concern when prescribing antibiotics for probable viral infections.” (Author Interpretation - Butler, 1998) | 1(21)  ^40^ | Very Low  Moderate concerns regarding methodological limitations (the data comes from a single study with moderate rigour), Moderate concerns regarding coherence ( the data comes from a single study and did not assess the phenomenon of interest in various contexts), Moderate concerns regarding adequacy ( the data is from a single study and small sample but data is not superficial), and Serious concerns regarding relevance (the data comes from a single study and focuses on only one URTI) |
| Goal | Barrier | Do what is clinically best for the patient and prescribe antibiotics | “Although a minority mentioned bacterial resistance as a potential problem, this was seen as a community issue whereas the general practitioners’ priority was the well being of the individual patient.” (Author Interpretation - Butler, 1998) | 4(49)  ^40,43,55,56^ | Low  No/Very minor concerns regarding methodological limitations, Moderate concerns regarding coherence (25% of the supporting data is vague and unclear), No/Very minor concerns regarding adequacy, and Moderate concerns regarding relevance (all studies are of only partial relevance to the review's population of interest (i.e., URTIs) but several URTIs are represented). |
|  | Barrier | “do something” for the patient, therefore the FPs prescribe antibiotics | “...I think doctors like to do something. You don't like to think there is nothing you can do, and there's nothing you can offer.” (Direct Quote - Dempsey, 2014) | 2(24)  ^44,56^ | Very Low  No/Very minor concerns regarding methodological limitations, Moderate concerns regarding coherence (50% of the data is vaguely described), Serious concerns regarding adequacy (while the finding is relatively simple, the data lacks some richness and comes from only two studies with few participants), and Serious concerns regarding relevance (the data is only of partial relevance to the review's population of interest (i.e., URTIs)). |
|  | Barrier | Patient satisfaction above all else therefore, prescribe antibiotics | “I think when I was a young fiery FP trainee I used to try and not give antibiotics and now I’m softening and I give antibiotics more than I used to . . . I’m quite well aware of the lack of firm evidence that antibiotics treat URTIs and that in terms of evidence based medicine we overprescribe antibiotics, but my own view is that I don’t really care . . . **your goals at the end of the conversation is for both you and the mother and the baby to be satisfied.”**(Direct Quote - Butler, 1998) | 1(21)  ^40^ | Very Low  Moderate concerns regarding methodological limitations (the data comes from a single study with moderate rigour), Moderate concerns regarding coherence (the data comes from a single study and did not assess the phenomenon of interest in various contexts), Moderate concerns regarding adequacy(the data is from a single study and a single setting but is not superficial), and Serious concerns regarding relevance (the data comes from a single study and focuses on one URTI) |
|  | Barrier | To not run over appointment time which leads to prescribing. | ‘… the pressure that patients put on locums to prescribe, and the pressure of time on the locum […] it is to do with the time that we have, so you’re paid by the hour. **You don’t necessarily want to run over […]**” (Direct Quote - Borek, 2022) | 1(19)  ^39^ | Very low  Moderate concerns regarding methodological limitations (because the data is from a single study with moderate rigour), No/Very minor concerns regarding coherence, Moderate concerns regarding adequacy(the data comes from a single study, small sample, but data is not superficial), and Serious concerns regarding relevance( the study contains data on conditions outside the inclusion criteria) |
|  | Enabler | Promote antibiotic stewardship and follow best practice | "I’m pretty conservative to use antibiotics. I try to practice evidence-based medicine and the evidence doesn’t really support antibiotic prescribing for upper respiratory tract infections." (Direct Quote - Patel, 2019) | 4(102)  ^40,51,52,54^ | High  No/Very minor concerns regarding methodological limitations, No/Very minor concerns regarding coherence, Minor concerns regarding adequacy (the study settings are similar but the data is not superficial), and No/Very minor concerns regarding relevance |
| Memory attention and decision processes | Barrier | Uncertainty when diagnosing URTIs can lead to inappropriate antibiotics prescription for URTIs | “The physician can never be 100% sure it's not a bacterial infection, so they worry about that. So there's some clinical uncertainty.” (Direct Quote - Dempsey, 2014)  “FPs acknowledged that there are guidelines on antibiotic prescribing but explained that there can be challenges when trying to decide on prescribing antibiotics, especially when neither the person’s condition is clear-cut nor the length of time that they have been having symptoms.” (Author Interpretation - O’Doherty, 2019) | 12(236)  ^40,41,44–46,49,52,53,56–58,61^ | High  No/Very minor concerns regarding methodological limitations, Minor concerns regarding coherence (threats present (ambiguous data) in <25% of the supporting data), Minor concerns regarding adequacy(the data comes from multiple studies in different settings and varying sample sizes and <25% of the supporting data is too superficial), and No/Very minor concerns regarding relevance |
|  | Enabler | FPs need a clear indication to prescribe antibiotics | “I feel like I need a clear indication. For strep, I have a positive strep because we have the test for that and pneumonia, I have a positive X-ray. … I like a clear indication for why I’m giving the antibiotics because of the side efects and the resistance.” (Direct Quote - Simeoni, 2022) | 4(89)  ^41,51,52,58^ | Moderate  No/Very minor concerns regarding methodological limitations, No/Very minor concerns regarding coherence, Moderate concerns regarding adequacy (the data comes from only a few studies or small studies and 25-50% of the supporting data is too superficial), and No/Very minor concerns regarding relevance |
|  | Neutral/Unclear | FPs consider clinical factors like clinical presentation, medical history, clinical risk, and age in there management and antibiotic prescribing decisions for URTIs | “The FPs' reasons for prescribing antibiotics were longer duration of the before-mentioned complaints (5-21 days while local therapy was used), severe complaints with pathological results on physical examination (e.g., localized sinus-related pain, purulent rhinorrhoea, mucoid swelling and affected general condition), and a high risk of complications of acute sinusitis.” (Author Interpretation - de Bock, 1994)  "If it’s (duration) longer than 7–10 days then it is likely that it is bacterial, so I may prescribe antibiotics. So, in that range, if I’ve been sick for 8– 9 days I will often times ask a clarifying question, do you think your symptoms are getting better, worse or same. And that can give me a little bit of an idea of the trajectory of the symptoms.” (Direct Quote - Patel, 2019)  “And … if I have met a patient who fits the criteria and if I have found something objective and precise … I… the most important thing for me is the clinical status.… How the patient is feeling, is affected… and so if I find something by objective examination, when I look at the throat, that … for example, I take no samples, I treat directly.” (Direct Quote - Tystrup, 2020) | 16(357)  ^39,40,42,43,45,50–54,56–60^ | N/A |
|  | Neutral/Unclear | FPs consider factors such as clinical presentation, medical history, clinical risk, guideline recommendations and clinical observations to guide diagnosis of URTIs | “Therefore, physicians instead relied on their clinical observations, which they believed to be sufficient in most cases, to make a proper diagnosis.” (Author Interpretation - Kaae, 2017)  “All FPs said that sore throat consultations were simple compared with other consultations and adherent FPs reported a targeted patient history and examination emphasizing the Centor criteria (Quotation E) while non-adherent FPs described a redundant patient history and examination as compared with guidelines (i.e. asking if a neighbour had tonsillitis or always examining lungs or vocal chords).” Author Interpretation - Hedin, 2014) | 13(260)  ^40,41,43,46,48,49,51,54–56,58–60^ | N/A |
| Environmental context and resources | Barrier | Lack of access to other healthcare or resources leads to more defensive prescribing | "Due to lack of lab facilities tests for patients in the facility it is difficult to diagnose the nature of infection whether it is bacterial or viral. In order to not take the risk, patient is thus given antibiotic" (Direct Quote - Kotwani, 2017) | 9(194)  ^45,48–50,52,55–57,60^ | High  No/Very minor concerns regarding methodological limitations, Minor concerns regarding coherence (threats present in <25% of the supporting data (some ambiguous data)), Minor concerns regarding adequacy(the data comes from multiple studies in different settings and varying sample sizes and <25% of the supporting data is too superficial), and Minor concerns regarding relevance (some of the supporting data (< 25%) is of partial relevance) |
|  | Barrier | Time pressure, work load or day of the week can influence FPs to prescribe antibiotics for URTIs | “But if it is an upper respiratory that probably looks viral but could deteriorate but coming up towards the end of the week, I would probably give them a deferred script” (Direct Quote - O’Doherty, 2019)  "I think the less time you have you are more likely to do a quick interview and it’s easy to provide an antibiotic and go to the next patient." (Direct Quote - Patel, 2019) | 13(293)  ^39–41,45,46,50–56,58^ | High  No/Very minor concerns regarding methodological limitations, No/Very minor concerns regarding coherence, Minor concerns regarding adequacy(the data comes from multiple studies in different settings and varying sample sizes and <25% of the supporting data is too superficial), and Minor concerns regarding relevance (less than 25% includes data that is partially relevant to the review) |
|  | Barrier | Workplace culture, environment or location can influence FPs to prescribe antibiotics for URTIs | “Participants’ relationships and communication with practices varied considerably. They reported no communication about practices’ initiatives or approaches related to prescribing and AMS[antimicrobial stewardship] (for example, antibiotic-related targets and priorities),...” (Author Interpretation - Borek, 2022) | 4(97)  ^39,50,53,58^ | High  No/Very minor concerns regarding methodological limitations, Minor concerns regarding coherence(threats (ambiguous data) present in <25% of the supporting data), Minor concerns regarding adequacy(the data comes from multiple studies in different settings and varying sample sizes and <25% of the supporting data is too superficial), and Moderate concerns regarding relevance( just over 25% of the supporting data is from partial relevance to the review). |
|  | Barrier | Lack of guidelines for antibiotic prescribing for URTIs to follow can increase inappropriate prescribing | “As no national clinical guidelines on ABs exist in Albania, the HCPs stated that their basis for AB knowledge and AB-related practices came from continuing educational activities, such as materials on the internet, visits from pharmaceutical company representatives, discussions with colleagues, and their formal education.” (Author Interpretation - Kaae, 2017) | 4(94)  ^41,47–49^ | Moderate  No/Very minor concerns regarding methodological limitations, Minor concerns regarding coherence, Moderate concerns regarding adequacy(from only a few studies or small studies and 25-50% of the supporting data is too superficial), and Minor concerns regarding relevance. |
|  | Barrier | FPs are incentivized by saving time and financial gains to prescribe antibiotics for URTIs by saving time and financial gains | “if you do it, you can see more patients, because you end the visit quicker instead of having a long discussion, trying to get their buy-in to not prescribe. So, actually, yes, in essence [there are time and financial incentives]; but not in the we-get-paid-to-use-this-drug-stuff-[way] .” (Direct Quote - Dempsey, 2014) | 1(12)^44^ | Low  (Moderate concerns regarding methodological limitations( the data comes from a single study with moderate rigour), No/Very minor concerns regarding coherence, Moderate concerns regarding adequacy(the data comes from a single study and setting but the data is not thin), and Minor concerns regarding relevance(some of the supporting data (< 25%) is of partial relevance)) |
|  | Barrier | Access to over-the-counter Antibiotics impede FP's ability to manage URTIs without prescribing | “Doctors routinely encounter patients who "either buy the same antibiotics on getting similar symptoms again or recommend/pass on the antibiotics they took at the time of their illness, to friends or relatives with similar symptoms" (Direct Quote - Kotwani, 2017) | 2(96)  ^47,50^ | Low  No/Very minor concerns regarding methodological limitations, No/Very minor concerns regarding coherence, Serious concerns regarding adequacy (the data comes from only a few studies or small studies and >50% of the supporting data is too superficial), and Serious concerns regarding relevance (some of the supporting data (25%-50%) comes from data of partial relevance; the finding is primary care model specific and may not generalize). |
|  | Barrier | Antibiotic prescribing for URTIs can increase due to excess antibiotic supply | "Medicine supply is erratic and irregular. There are instances when a certain drug is not in supply for many months while sometimes there will be an oversupply. Authorities put constant pressure on doctors to prescribe the drugs if they are abundant in supply and to exhaust them before their expiry date" (Direct Quote - Kotwani 2017) | 1(45)  ^50^ | Low  No/Very minor concerns regarding methodological limitations, No/Very minor concerns regarding coherence, Serious concerns regarding adequacy(one study offering thin data), and Moderate concerns regarding relevance(the data included a condition outside of our inclusion criteria but the data was reported separately). |
|  | Barrier | Cost of consultation pressures FPs to prescribe so the patient leaves with something | “This is another ethical challenge which FPs face in OOH settings; they indicated that some FPs find it difficult to let a private patient leave the consultation without a prescription for an antibiotic because they are paying a fee.” (Direct Quote -) O’Doherty, 2019 | 1(13)  ^53^ | Very Low  No/Very minor concerns regarding methodological limitations, Moderate concerns regarding coherence (lack of ability to assess presence of contradictory data and contextual variation), Serious concerns regarding adequacy (the data is thin and comes from a single study), and Serious concerns regarding relevance (the finding and data are related to the type of primary care funding model in place and may not be relevant to other types of primary care models). |
|  | Barrier | Antibiotic prescribing for UTRIs can increase due to their designation as reimbursable drugs | “Study participants from both countries reported that the addition of an antibiotic to the list of medications reimbursable by state or health insurance makes it more accessible and triggers an increase in its use.” (Author Interpretation - Jaruseviciene 2013) | 1(51)  ^47^ | Very Low  Moderate concerns regarding methodological limitations (the data is from a single study with moderate rigour), No/Very minor concerns regarding coherence, Serious concerns regarding adequacy (the data is thin and comes from a single study), and Serious concerns regarding relevance (the data comes from a single study and is related to the primary care funding model and may not be relevant to other types of models). |
|  | Barrier | A community’s health and socioeconomic status can influence FPs to prescribe antibiotics for URTIs | “I have great doubt, I mean, one of the, I mean, I confess the issue for me is generalisability of some of the work that’s been done. You can’t deny the differences between the comfortable middle class patients in the South [of England] and inner city Glasgow. I mean the thing that did it for me—I saw a slide on Aborigines and they’re in appalling social conditions, and they still got complications like mastoiditis. And I know you might say, “Oh well it’s genetics,” but honestly I believe that a lot of it is due to poor nutrition, poor housing conditions, and overcrowding. So I must admit these factors affect my prescribing.” (Direct Quote - Kumar, 2003) | 2(52)  ^51,52^ | Very Low  Minor concerns regarding methodological limitations (data comes from only two studies and one study has moderate rigour), Serious concerns regarding coherence (the data from one of the two studies is unclear and may not support the review finding), Serious concerns regarding adequacy(the data is thin and comes from only two studies), and Serious concerns regarding relevance (the data comes from only two studies and one study is only related to a single URTI). |
|  | Barrier | Lack of political leadership or policies in place to help reduce unnecessary prescribing | “Study participants from both countries emphasized the necessity of political measures aimed at limiting antibiotic over-use, expressing strong doubt about whether interventions oriented exclusively towards physicians can be effective without political support” (Author Interpretation - Jaruseviciene, 2013) | 1(51)  ^47^ | Very Low  Moderate concerns regarding methodological limitations (the data is from a single study with moderate rigour), No/Very minor concerns regarding coherence, Moderate concerns regarding adequacy (the data is from a single study and the data is thin but is also context-specific), and Serious concerns regarding relevance (the data comes from a single study that included two countries and the finding may be country-specific and may not be relevant globally) |
|  | Barrier | Time of year may increase antibiotic prescribing | Respondents reported that antibiotics are prescribed more often during busy farming seasons, with many township hospital physicians mentioning that injectable antibiotics are used more frequently at this time of year. (Author Interpretation – Shen, 2023) | 1(30)  ^57^ | Very low  No/Very minor concerns regarding methodological limitations, Serious concerns regarding coherence (the data comes from a single study and did not assess for the presence of contradictory data), Serious concerns regarding adequacy(the data comes from a single study and does not include a sample of FPs that would be considered representative of all general clinical practice in the study location), and Serious concerns regarding relevance (the findings are specific to the location where the research was conducted and may not apply to other locations/contexts). |
|  | Enabler | Having educational resources for FPs or patients has helped FPs reduce prescribing | “I think it would be great [I have] the one that says, Antibiotics don't work for colds and flu. Got that right next to where my head is, so people, when they're looking at me, they see the thing saying, Antibiotics are not for you” (Author Interpretation - Dempsey, 2014) | 3(49)  ^41,44,58^ | High  No/Very minor concerns regarding methodological limitations, No/Very minor concerns regarding coherence, Minor concerns regarding adequacy(the data comes from multiple studies in different settings and varying sample sizes and <25% of the supporting data is too superficial), and No/Very minor concerns regarding relevance. |
|  | Enabler | Having clear guidelines for antibiotic prescribing for URTIs can reduce unnecessary prescribing | “Physicians repeatedly mentioned the Choosing Wisely campaign as a supportive resource that helped them achieve guideline-concordant antibiotic prescribing.” (Author Interpretation - Simeoni, 2022) | 2(37)  ^41,58^ | Moderate  No/Very minor concerns regarding methodological limitations, No/Very minor concerns regarding coherence, Moderate concerns regarding adequacy (the data comes from only a few studies or small studies and 25-50% of the supporting data is too superficial), and No/Very minor concerns regarding relevance). |
|  | Enabler | Access to healthcare and resources helps reduce prescribing | “In my own practice, there’s a level of trust, and there’s also accessibility. So, if I instruct the patient with, I don’t think you need an antibiotic right now but I’m here all week, you know how to get a hold of me, you can always come back… “(Direct Quote - Simeoni, 2022) | 2(28)  ^55,58^ | Low  (Minor concerns regarding methodological limitations (one study has rated as having moderate methodological rigour), Moderate concerns regarding coherence (lack of ability to assess contradictory data), Serious concerns regarding adequacy (data comes from a single study), and No/Very minor concerns regarding relevance) |
|  | Enabler | Having nurses in the clinics to triage patients can help reduce antibiotic prescribing for URTIs | “And really good education from the nurses on triage, I think, could actually do a good job of not even bothering to bring these people in.” (Direct Quote - Dempsey, 2014) | 1(12)  ^44^ | Very Low  Moderate concerns regarding methodological limitations (the data was from a single study with moderate rigour), No/Very minor concerns regarding coherence, Moderate concerns regarding adequacy (the data is from a single study, small sample but is not thin), and Moderate concerns regarding relevance (the data comes from a single study that focuses on one URTI). |
|  | Neutral/ Unclear | Costs associated with care and antibiotics influence antibiotic prescribing for URTIs | “FPs mentioned how prescribing pattern might be influenced depending on whether the patient is paying or has access to free healthcare.” (Author Interpretation - O’Doherty, 2019) | 4(41)  ^44,48,52,53^ | N/A |
|  | Neutral/ Unclear | Access to testing and results influence antibiotic prescribing for URTIs | “Near-patient C-reactive protein (CRP) test was regarded by the FPs an important tool to differentiate between bacteria and virus.” (Author Interpretation - Tystrup, 2020) | 2(41)  ^52,60^ | N/A |
| Social Influences | Barrier | Influenced negatively by or lacking support from colleagues, supervisors or clinic to not prescribe antibiotics unnecessarily for URTIs | “I saw a patient today who has COPD and you look back and see that’s what they do and I’m more inclined to prescribe antibiotics just in case because that’s what they do, rather than making my own judgement, I’m just following what the practice are doing.” (Direct Quote - Borek, 2022)  “I do know one supervisor in particular will give his patients antibiotics even for something that sounds very viral, and therefore when I see his patients, I feel I’m expected to do that as well, because his patients have been seeing him for many years. So they expect it too, so I’m definitely more likely to give his patients antibiotics even when I don’t think it’s justified.” (Direct Quote - Dallas, 2013) | 8(157)  ^39,41,44,46,49,52,57,58^ | High  No/Very minor concerns regarding methodological limitations, Minor concerns regarding coherence( some of the data (<25%) was ambiguous), Minor concerns regarding adequacy(the data comes from multiple studies in different settings and varying sample sizes and <25% of the supporting data is too superficial), and Minor concerns regarding relevance (some of the supporting data (< 25%) is of partial relevance( i.e., one of the conditions in a study is outside of our scope) |
|  | Barrier | Lack of patient relationship, prior knowledge of patient or patients culture/history can increase prescribing | “If someone comes in really unwell—high temperature and looks toxic—and they’ve hardly ever been in to see me before, then I take them pretty seriously. They must have managed a lot of sore throats at home so it must be bad if they’ve come in, so I’m more ready to prescribe an antibiotic.” (Direct Quote - Kumar, 2003)  “The problem with a walk-in, you don’t establish a relationship with the patient that you get to see once. They see you as a walk-in doctor. ‘I’m only going to see you once, I don’t really care what you say.’ You know, that kind of thing. ‘I’m here for that, this is what I want, this is what my doctor would have given me’, you know. And I think that’s where the major difference is.” (Direct Quote - Simeoni, 2022) | 10(240)  ^39,40,44,45,51,54,56–59^ | High  No/Very minor concerns regarding methodological limitations, Minor concerns regarding coherence (ambiguous data present in <25% of the supporting data), No/Very minor concerns regarding adequacy, and Minor concerns regarding relevance(some of the supporting data (< 25%) is of partial relevance (one study included a condition outside of our inclusion criteria) |
|  | Barrier | FPs report that patients do not understand that antibiotics will not work, they do not have the capacity to understand or not perceived as capable to take care of themselves or kid | “If they don’t understand that then unwittingly I would say, well I do not agree but since you say (so), I will give it to you…” (Direct Quote - Fletcher-Lartey, 2016)  “Other general practitioners described similar tensions when patients reattended with sore throat or when patients were unable to understand “complex explanations”—for example, because of lack of English language.” (Author Interpretation - Kumar, 2003) | 5(118)  ^40,44,45,51,53^ | High  No/Very minor concerns regarding methodological limitations, No/Very minor concerns regarding coherence, No/Very minor concerns regarding adequacy, and No/Very minor concerns regarding relevance |
|  | Barrier | FPs prescribe antibiotics for URTIs to meet patient expectation or demand (real or perceived) for antibiotics or for patient satisfaction | “General practice medicine is never just they come in, they have a fever, they must be treated. It's so-and-so doesn’t have a lot of money who’s kind of saved up for the appointment … They’ve taken time out of their busy schedule to come and see you, they’ve had to wait an hour and a half because you were running late … it’s all those other things and they’ve come in saying ‘all I need is my antibiotic script.” (Direct Quote - Dallas, 2014)  “You don't want people to feel like they're getting pre judged as not sick, because then they're going to come in even more defensive about BEING sick I’m not sure I'd want somebody that sort of primed to think they re not going to get antibiotics, because they're going to get more geared up.” (Direct Quote - Dempsey, 2014)  “I have no idea who does it because they won’t tell me, but I’m sure a lot of people do that. A lot of people go to several doctors to try and get antibiotics. They just want a prescription. There’s a lot of pressure on FPs, I know that.” (Direct Quote - Fletcher-Lartey, 2016)  ““There were so many times [in a walk-in] where it was like, you know what, why am I going through all this spiel. Because, at the end of it, after spending 15 min with the parents or with the patient, they’re just going to say, so what antibiotics are you going to give me.” (Direct Quote - Simeoni, 2022) | 21(436)  ^39–46,48–59,61^ | High  Minor concerns regarding methodological limitations (<25% of the supporting data comes from studies with low methodological rigour), No/Very minor concerns regarding coherence, No/Very minor concerns regarding adequacy, and Minor concerns regarding relevance(some of the supporting data (< 25%) is of partial relevance (including a condition that is outside our scope) |
|  | Barrier | FPs reported external sources like pharmaceutical companies, policy makers, and auditors influence them to prescribe | "... Authorities put constant pressure on doctors to prescribe the drugs if they are abundant in supply and to exhaust them before their expiry date” (Direct Quote - Kotwani, 2017) | 2(96)  ^47,50^ | Low  No/Very minor concerns regarding methodological limitations, No/Very minor concerns regarding coherence, Moderate concerns regarding adequacy (the data is from a small number of studies, and may not be representative of all clinicians), and Serious concerns regarding relevance ( half of the data may be of indirect to this review(they include a condition outside of our criteria) and this finding may be relevant to specific healthcare models). |
|  | Enabler | FPs noted that patients don't necessarily want antibiotics or expect them, and/or the demand for them has decreased or this dissatisfaction didn't bother them | “So the patients get quite a diverse range of opinions, and they may have been treated with penicillin two or three times and then they come and see me and I explain why they don’t need antibiotics. I still find people reasonably accepting. I think most of them realize before they see me that that is probably what they are going to be told.” (Direct Quote - Kumar, 2003)  "For most patients who come in, their goal is not to get an antibiotic, they want to make sure they are not severely sick or messing around or delay treatment, so if I assure them in such cases, they are pretty satisfied." (Direct Quote - Patel, 2019) | 7(153)  ^40,44,46,48,49,51,54^ | High  No/Very minor concerns regarding methodological limitations, No/Very minor concerns regarding coherence, No/Very minor concerns regarding adequacy, and No/Very minor concerns regarding relevance |
|  | Enabler | FPs said that having a good doctor patient relationship/knowledge of the patient helped them not prescribe antibiotics unnecessarily for URTIs | "Over time I think patients trust you and most of my patients have been with me for 15 years and I think they trust me so maybe it’s different than when I started out and I tried to tell them that it was viral and they would roll their eyes till the back of their heads, and now over time they know that I’m not going to withhold treatment without a reason and they kind of go with what I’m suggesting." (Direct Quote - Patel, 2019) | 2(49)  ^54,58^ | Moderate  (No/Very minor concerns regarding methodological limitations, No/Very minor concerns regarding coherence, Moderate concerns regarding adequacy(the data is not superficial but comes from only two studies from North America), and No/Very minor concerns regarding relevance) |
|  | Enabler | Colleagues, supervisor or clinic support to help not prescribe unnecessarily | “Initially you didn’t know how to argue with a patient and then you just give in and give antibiotics. Then after having a chat with my supervisor, he said you could try doing it this way, and that seems to work quite well.” (Direct Quote - Dallas, 2014) | 2(42)  ^41,46^ | Moderate  (No/Very minor concerns regarding methodological limitations, No/Very minor concerns regarding coherence, Moderate concerns regarding adequacy(the data is not superficial but comes from only two studies with smaller sample sizes), and No/Very minor concerns regarding relevance) |
|  | Enabler | FPs report that some policies, local prescribing advisors helped them reduce prescribing | “They identified external pressures, such as research, local prescribing advisors, and national reports, that had influenced them to reduce antibiotic prescribing.” (Author Interpretation - Kumar, 2003) | 1(40)  ^51^ | Very Low  Moderate concerns regarding methodological limitations (data comes from a single study with moderate rigour), No/Very minor concerns regarding coherence, Moderate concerns regarding adequacy (the data is based on a single study but the data is not superficial), and Serious concerns regarding relevance ( the data comes from a single study that focused on only one URTI and the finding may be country-specific). |
|  | Neutral/  Unclear | FPs highlight that external sources like pharmaceutical companies, policy makers, and auditor influence prescribing | “Study participants reported that pharmaceutical companies influence antibiotic prescribing in two ways: directly through visits to medical practitioners and indirectly through support of continuing medical education for physicians.” (Author Interpretation - Jaruseviciene, 2013) | 4(140)  ^47,48,50,51^ | N/A |
|  | Neutral/  Unclear | Importance of patient expectations and satisfaction on antibiotic prescribing behaviour | “Unless you find out what they’re really in there for and how strongly they expect a prescription, I think you’re not going to deal with the consultation as effectively.” (Direct Quote - Butler, 1998) | 5(134)  ^39,40,51,54,55^ | N/A |
|  | Neutral/  Unclear | Colleagues can significantly influence antibiotic prescribing decisions for URTIs | “… where they [the registrar] really like the supervisor, really respect them, and just do whatever they do and don’t think for themselves.” (Direct Quote - Dallas, 2014)  “Such practitioners learn from the prescribing patterns of doctors in cities and practice in their native villages or in slum areas of the city” (Author Interpretation - Kotwani, 2017) | 5(107)  ^41,44,48,50,54^ | N/A |
| Emotion | Barrier | Fatigue or lack of enthusiasm can influence FPs to prescribe antibiotics unnecessarily for URTIs | “Consequently, some cited being too busy or tiredness as reasons for prescribing.” (Author Interpretation - Dallas, 2014) | 5(78)  ^40,41,55,56,58^ | High  (No/Very minor concerns regarding methodological limitations, No/Very minor concerns regarding coherence, Minor concerns regarding adequacy(the data comes from multiple studies in different settings and varying sample sizes and <25% of the supporting data is too superficial), and No/Very minor concerns regarding relevance) |
|  | Barrier | Uncertainty in URTI diagnosis or management can make FPs frustrated or overwhelmed | “In the focus groups the physicians described AMS [acute maxillary sinusitis] as a difficult diagnosis and they sometimes faced the uncertainty with frustration.” (Author Interpretation - Varonen, 2004) | 2(37)  ^41,61^ | Moderate  (No/Very minor concerns regarding methodological limitations, No/Very minor concerns regarding coherence, Serious concerns regarding adequacy(only two studies, two settings with small sample sizes and supporting data is thin (>50%), and No/Very minor concerns regarding relevance) |
|  | Barrier | Prescribing antibiotics for URTIs did not make FPs uncomfortable | “I don’t feel uncomfortable because I’m prescribing; I feel uncomfortable sometimes for not prescribing.” (Direct Quote - Kumar, 2003) | 2(60)  ^51,61^ | Moderate  (No/Very minor concerns regarding methodological limitations, No/Very minor concerns regarding coherence, Moderate concerns regarding adequacy(only two studies, two countries, and 50% of data is thin), and No/Very minor concerns regarding relevance) |
|  | Barrier | FPs may prescribe antibiotics if they have a negative "gut-feeling" about a patient's condition | “In this process gut feeling came into play by stating things like “simply knowing the story of the patient” and a “certain feeling… If that feeling was not good, there was a tendency to prescribe antibiotics…”  (Author interpretation – Schubert, 2023) | 1(12)  ^56^ | Low  No/Very minor concerns regarding methodological limitations, Serious concerns regarding coherence (the data comes from a single study and did not assess for contradictory data or variation in different contexts), Serious concerns regarding adequacy (the data comes from a single study, lacks richness and does not come from a sufficiently representative sample of physicians), and No/Very minor concerns regarding relevance. |
|  | Barrier | Prescribing antibiotics for URTIs can happen when FPs feel under pressure | "... So now I prescribe when I feel under pressure ..."(Direct Quote - Kumar, 2003) | 1(40)  ^51^ | Very low  Moderate concerns regarding methodological limitations (he data is from a single study with moderate rigour), Serious concerns regarding coherence (no ability to assess presence of contradictory data), Serious concerns regarding adequacy (he data comes from only a few studies or small studies and >50% of the supporting data is too superficial), and Serious concerns regarding relevance (the data comes from a single study and focuses on only one URTI) |
|  | Enabler | Prescribing antibiotics made physicians feel compromised or used | “It does make me feel uncomfortable. I do feel as though I’ve been slightly used. Sometimes slightly abused as well.” | 1(21)  ^40^ | Very Low  Moderate concerns regarding methodological limitations ( the data is from a single study with moderate rigour), No/Very minor concerns regarding coherence, Serious concerns regarding adequacy ( the data comes from only a few studies or small studies and >50% of the supporting data is too superficial), and Serious concerns regarding relevance (the data comes from a single study that focuses on one URTI) |
|  | Enabler | Not prescribing antibiotics unnecessarily for URTIs lead to FP satisfaction | “Obviously if I prescribe antibiotics then the patient is happy, but if they accept a rational explanation as to why they don’t want antibiotics and seem happy enough with that, then that’s equally, even more satisfying really.” (Direct Quote - Butler, 1998) | 1(21)  ^40^ | Very Low  Moderate concerns regarding methodological limitations (the data is from a single study with moderate rigour), Moderate concerns regarding coherence ( the data is not thin but comes from a single study and the sample of FPs is likely fully representative of all FPs), Moderate concerns regarding adequacy (the data comes from a single study, a single setting), and Serious concerns regarding relevance (the data is focused on only one condition instead of all or several URTIs). |
| Behavioural Regulation | Enabler | Delayed prescribing | “About half said they often gave patients a prescription but asked them to wait a few days before deciding  to take the medicine (a delayed prescription strategy)” (Author Interpretation - Butler, 1998)  “I think sometimes the compromise there is the deferred script but you say ‘I don’t think you need to go on something right away. Hold off, there is a prescription for [name of an antibiotic], three times daily for 5 days but I would be hoping you don’t need to fill it’” (Direct Quote - O’Doherty, 2019). | 7(146)  ^40,45,51,53,55,56,58^ | High  Minor concerns regarding methodological limitations, No/Very minor concerns regarding coherence, No/Very minor concerns regarding adequacy, and No/Very minor concerns regarding relevance. |
|  | Enabler | Patient education | "Consumer education (discussion/explanation) was the most common strategy reported by participants to manage patients’ expectations and demands for antibiotics." (Author Interpretation - Fletcher-Lartey, 2016)  "... many physicians mentioned using patient-facing resources, such as Choosing Wisely pamphlets [17], and symptomatic treatment as an alternative to an antibiotic, as these tools helped reinforce the physician's decision, and the patient would leave feeling that their symptoms were being taken seriously. " | 8(185)  ^39,40,44,45,51,52,54,58^ | High  No/Very minor concerns regarding methodological limitations, No/Very minor concerns regarding coherence, No/Very minor concerns regarding adequacy, and Minor concerns regarding relevance (some of the supporting data (< 25%) is of partial relevance). |
|  | Enabler | Feedback on prescribing | “I remember I was surprised, disappointed, and it definitely, kind of, firmed up my resolve to be a little bit more restrictive in my prescribing [when received feedback about their antibiotics prescribing]. I can tell you that the information I did receive changed my practice to some extent. So I did not feel like that was inappropriate. I mean, being compared to your peers is always motivating. Because when you compare it to a study, you can always say, well, my patient doesn’t meet this study criteria. But I can’t convince myself that, oh, I’m seeing sicker people than my neighbouring physician…… it’s [the feedback] just made me a little more resolute in saying, no, this is viral, no antibiotic.” (Direct Quote - Simeoni, 2022) | 2(32)  ^44,58^ | Moderate  No/Very minor concerns regarding methodological limitations, No/Very minor concerns regarding coherence, Moderate concerns regarding adequacy (the data comes from only two studies and may not represent all clinicians, but the data is not thin), and No/Very minor concerns regarding relevance |
|  | Enabler | The “wait and see” approach | “... But I always say, if you get worse in any way, if you develop a fever after today, I want you to call me and come back and be reassessed to see if I change my treatment plan.” (Direct Quote - Simeoni, 2022) | 2(41)  ^40,58^ | Moderate  No/Very minor concerns regarding methodological limitations, No/Very minor concerns regarding coherence, Moderate concerns regarding adequacy (data is not thin but only from two studies, both in first world countries), and No/Very minor concerns regarding relevance |
|  | Enabler | Team support | “Initially you didn’t know how to argue with a patient and then you just give in and give antibiotics. Then after having a chat with my supervisor, he said you could try doing it this way, and that seems to work quite well.” (Direct Quote - Dallas, 2014) | 2(29)  ^41,44^ | Moderate  (No/Very minor concerns regarding methodological limitations, No/Very minor concerns regarding coherence, Moderate concerns regarding adequacy (the data comes from only a couple studies and is fairly superficial), and No/Very minor concerns regarding relevance) |
|  | Enabler | External antibiotic regulation or oversight | "I know the pharmacy people track what we prescribe specifically, whether its generic or name brand they track these measures and so they could track who[is] prescribing antibiotics. Having evidence, like the number of times that you prescribed antibiotics; these are the cases where you did it.” (Direct Quote - Dempsey, 2014) | 2(63)  ^44,47^ | Moderate  (No/Very minor concerns regarding methodological limitations, No/Very minor concerns regarding coherence, Moderate concerns regarding adequacy (data is not thin but comes from only a couple studies), and No/Very minor concerns regarding relevance) |
|  | Enabler | Guidelines and clinical tools | "When we had [an earlier form of electronic clinical decision support], that made it really easy, in my opinion, to be able to more easily flow through these visits because it had these prompts that were a little reminder not only did it have the reminders, but then it had all the kind of symptomatic treatment stuff that you could just print out with a click of a button, like, give them the cough syrup and the Tylenol and the this and the that and the patient handouts on it I really liked that form, and I wish it hadn’t gone away.” (Direct Quote - Dempsey, 2014) | 4(60)  ^39,41,44,52^ | Moderate  (No/Very minor concerns regarding methodological limitations, No/Very minor concerns regarding coherence, No/Very minor concerns regarding adequacy, and Moderate concerns regarding relevance(25%-50% of supporting data has inclusion criteria that is of partial relevance)) |
|  | Enabler | Diagnostic Tests | “...she took throat swabs when patients appeared toxic. Her reasons for doing so were to manage her own and the patients’ uncertainty, to delay or prevent antibiotic prescription, and to support her explanation that such symptoms could be caused by a virus.” | 4(95)  ^49,51,55,59^ | Low  No/Very minor concerns regarding methodological limitations, No/Very minor concerns regarding coherence, Moderate concerns regarding adequacy (the data is not thin but comes from only four studies), and Serious concerns regarding relevance (the majority of the data (>50%) is only partially relevant to this review's population of interest (i.e., URTI's).). |
|  | Enabler | Over-the-counter prescription pad | “The second clinician used the over-the-counter prescription pad to put the encounter: ‘in a positive light. You can spin things anyway, Well, the really good news is, you don't actually need to take an antibiotic. Your body can fight this off, we can help it, you know, with these ways.’” (Direct Quote - Dempsey, 2014). | 1(12)  ^44^ | Very Low  Moderate concerns regarding methodological limitations (the data is from a single study of moderate rigour), No/Very minor concerns regarding coherence, Serious concerns regarding adequacy (the data comes from only a single study and all of the supporting data is too superficial), and Moderate concerns regarding relevance (the data focuses on only one URTI) |
|  | Enabler | Making it uncomfortable to ask for antibiotics with signage | "Making the patient uncomfortable or trying to convince patients they had wasted the doctor’s time were considered legitimate strategies for reducing antibiotic prescribing." (Author Interpretation - Kumar, 2003) | 1(40)  ^51^ | Very Low  Moderate concerns regarding methodological limitations (the data is from a single study with moderate rigour), No/Very minor concerns regarding coherence, Moderate concerns regarding adequacy (the data is not thin but comes from a single study), and Moderate concerns regarding relevance (the data comes from a single study and focuses on only one URTI). |
|  | Enabler | Falsification of medical records | “The effort to avoid potential problems with external auditors and at the same time to follow clinical guidelines sometimes results in the falsification of medical records: the prescription is registered, but in fact is not given to the patient.” (Author Interpretation - Jarvusciene, 2013) | 1(51)  ^47^ | Very Low  Moderate concerns regarding methodological limitations (data comes from a single study with moderate rigour), No/Very minor concerns regarding coherence, Moderate concerns regarding adequacy (because the data comes from a single study and is thin), and Serious concerns regarding relevance (the data comes from a single study and may be country-specific). |
|  | Enabler | Longer appointment times | “Thus, some requested longer appointments or catch-up slots to ensure sufficient time to provide good-quality care (for example, discussing antibiotics and safety-netting).” (Author Interpretation - Borek, 2022) | 1(19)  ^39^ | Very low  (Moderate concerns regarding methodological limitations (the data comes from a single study with moderate rigour), No/Very minor concerns regarding coherence, Moderate concerns regarding adequacy (data is superficial and comes from a single study), and Moderate concerns regarding relevance (the data comes from a study that includes a condition outside of the scope of this review)) |
|  | Enabler | Locums specific methods to improve prescribing within a new practice | “Participants suggested that working in one local area and regular, longer-term practices helped to minimize this challenge, and that adopting similar guidelines and approaches would make appropriate prescribing easier for locums” (Author Interpretation - Borek, 2022) | 1(19)  ^39^ | Very low  Moderate concerns regarding methodological limitations (data comes from a single study with moderate rigour), No/Very minor concerns regarding coherence, Moderate concerns regarding adequacy (is not thin but comes from a single study), and Moderate concerns regarding relevance (data comes from a single study with a condition outside of our scope)) |
